# Optimal vaccination: various (counter) intuitive examples

**DOI:** 10.1007/s00285-022-01858-5

**Published:** 2023-01-10

**Authors:** Jean-François Delmas, Dylan Dronnier, Pierre-André Zitt

**Affiliations:** 1 CERMICS, Ecole des Ponts, Champs-sur-Marne, France; 2 Univsersité de Neuchâtel, Neuchâtel, Switzerland; 3 LAMA, Univsersité Gustave Eiffel, Champs-sur-Marne, France

**Keywords:** Kernel operator, Vaccination strategy, Effective reproduction number, Multi-objective optimization, Pareto frontier, 92D30, 47B34, 47A10, 58E17, 34D20

## Abstract

In previous articles, we formalized the problem of optimal allocation strategies for a (perfect) vaccine in an infinite-dimensional metapopulation model. The aim of the current paper is to illustrate this theoretical framework with multiple examples where one can derive the analytic expression of the optimal strategies. We discuss in particular the following points: whether or not it is possible to vaccinate optimally when the vaccine doses are given one at a time (greedy vaccination strategies); the effect of assortativity (that is, the tendency to have more contacts with similar individuals) on the shape of optimal vaccination strategies; the particular case where everybody has the same number of neighbors.

## Introduction

### Motivation

The basic reproduction number, denoted by $R_0$, plays a fundamental role in epidemiology as it determines the long-term behavior of an epidemic. For a homogeneous model, it is defined as the number of secondary cases generated by an infected individual in an otherwise susceptible population. When this number is below 1, an infected individual causes less than one infection before its recovery in average; the disease therefore declines over time until it eventually dies out. On the contrary, when the reproduction number is greater than 1, the disease invades the population. It follows from this property that a proportion equal to $1 - 1/R_0$ of the population should be immunized in order to stop the outbreak. We refer the reader to the monograph of Keeling and Rohani (2008) for a reminder of these basic properties on the reproduction number.

In the heterogeneous generalization of classical compartmental models (Lajmanovich and Yorke 1976; Beretta and Capasso 1986; Delmas et al. 2022a), the population is stratified into homogeneous groups sharing the same characteristics (*e.g.*, time to recover from the disease and interaction with the other groups). For these so-called metapopulation models, it is still possible to define a meaningful reproduction number $R_0$, as the number of secondary cases generated by a typical infectious individual when all other individuals are uninfected (Diekmann et al. 1990). The reproduction number can then be identified as the spectral radius of the so-called next-generation matrix (Van Den Driessche and Watmough 2002). This encompasses SIS, SIR and SEIR epidemic models; see Section 2 in (Delmas et al. 2022b) for a discussion. With this definition, it is still true that the outbreak dies out if $R_0$ is smaller than 1 and invades the population otherwise; see Thieme (1985); Hethcote and Thieme (1985); Van Den Driessche and Watmough (2002); Thieme (2011); Delmas et al. (2022a) for instance.

Suppose now that we have at our disposal a vaccine with *perfect efficacy*, that is, vaccinated individuals are completely immunized to the disease. After a vaccination campaign, let $\eta$ denote the proportion of **non-vaccinated** individuals in the population: in inhomogeneous models, $\eta$ depends *a priori* on the group as different groups may be vaccinated differently. We will call $\eta$ a *vaccination strategy*. For any strategy $\eta$, let us denote by $R_e(\eta )$ the corresponding reproduction number of the non-vaccinated population, also called the *effective reproduction number*. In the metapopulation model, it can also be expressed as the spectral radius of the effective next generation matrix; see Equation (5) below. The choice of $\eta$ naturally raises a question that may be expressed as the following informal constrained optimization problem:

1 $$\begin{aligned} {\left\{ \begin{array}{ll} {\textbf {Minimize: }} &{} \text {the quantity of vaccine to administrate} \\ {\textbf {subject to: }} &{} \text {herd immunity is reached, that is},~R_e\le 1. \end{array}\right. } \end{aligned}$$

For practical reasons, we will instead look at the problem the other way around. If the vaccine is only available in limited quantities, the decision makers could try to allocate the doses so as to maximize efficiency; a natural indicator of this efficiency is the effective reproduction number. This reasoning leads to the following constrained problem:

2 $$\begin{aligned} {\left\{ \begin{array}{ll} {\textbf {Minimize: }} &{} \hbox { the effective reproduction number~}\ R_e \\ {\textbf {subject to: }} &{} \text {a given quantity of available vaccine.} \end{array}\right. } \end{aligned}$$

In accordance with (Delmas et al. 2021b), we will denote by $R_{e\star }$ the value of this problem: it is a function of the quantity of available vaccine. The graph of this function is called the *Pareto frontier*. In order to measure how bad a vaccination strategy can be, we will also be interested in maximizing the effective reproduction number given a certain quantity of vaccine:

3 $$\begin{aligned} {\left\{ \begin{array}{ll} {\textbf {Maximise: }} &{} \hbox { the effective reproduction number~}\ R_e \\ {\textbf {subject to: }} &{} \text {a given quantity of available vaccine.} \end{array}\right. } \end{aligned}$$

The value function corresponding to this problem is denoted by $R_e^\star$ and its graph is called the *anti-Pareto frontier*. We will quantify the “quantity of available vaccine” for the vaccination strategy $\eta$ by a cost $C(\eta )$. Roughly speaking the “best” (resp. “worst”) vaccination strategies are solutions to Problem (2) (resp. Problem (3)). Still following Delmas et al. (2021b), they will be called *Pareto optimal* (resp. *anti-Pareto optimal*) strategies.

The problem of optimal vaccine allocation has been studied mainly in the metapopulation setting where the population is divided into a finite number of subgroups with the same characteristics. Longini, Ackerman and Elverback were the first interested in the question of optimal vaccine distribution given a limited quantity of vaccine supply (Longini et al. 1978). Using the concept of next-generation matrix introduced by Diekmann, Heesterbeek and Metz Diekmann et al. (1990), Hill and Longini reformulated this problem thanks to the reproduction number (Hill and Longini 2003). Several theoretical and numerical studies followed focusing on Problem (1) and/or Problem (2) in the metapopulation setting (Goldstein et al. 2010; Poghotanyan et al. 2018; Duijzer et al. 2018; Hao et al. 2019). We also refer the reader to the introduction of Delmas et al. (2021b) for a detailed review of the bibliography.

In two previous works (Delmas et al. 2021b, a), we provided an infinite-dimensional framework generalizing the metapopulation model where Problems (2) and (3) are well-posed, justified that the optimizers are indeed Pareto optimal and studied in detail the Pareto and anti-Pareto frontiers. Since there is no closed form for the effective reproduction number, Problems (2) and (3) are hard to solve in full generality: our goal here is to exhibit examples where one can derive analytic expressions for the optimal vaccination strategies. The simple models we study give a gallery of examples and counter-examples to natural questions or conjectures, and may help understanding common rules of thumb for choosing vaccination policies. We will in particular be interested in the following three notions.

(i) **Greedy parametrization of the frontiers**. For the decision maker it is important to know if global optimization and sequential optimization are the same as one cannot unvaccinate people and redistribute the vaccine once more doses become available. More precisely, there is a natural order on the vaccination strategies: let us write $\eta '\le \eta$ if all the people that are vaccinated when following the strategy $\eta$ are also vaccinated when following the strategy $\eta '$. Let $\eta$ be an optimal solution of (2) for cost $c=C(\eta )$, that is, $R_e(\eta ) = R_{e\star }(c)$. If, for $c'>c$, we can find a strategy $\eta '\le \eta$ such that $R_e(\eta ') = R_{e\star }(c')$, then the optimization may be, at least in principle, found in a greedy way: giving sequentially each new dose of vaccine so as to minimize $R_e$ gives, in the end, an optimal strategy for any quantity of vaccine. By analogy with the corresponding notion for algorithms we will say in this case that there exists a *greedy parametrization* of the Pareto frontier. The existence of such a greedy parametrization was already discussed by Cairns in Cairns (1989) and is examined for each model throughout this paper.
(ii) **Assortative/Disassortative network**. The second notion is a property of the network called *assortativity*: a network is called assortative when the nodes tend to attach to others that are similar in some way and *disassortative* otherwise. The assortativity or disassortativity of a network is an important property that helps to understand its topology. It has been oberved that social networks are usually assortative while biological and technological networks are disassortative, see for example (Newman 2002). The optimal vaccination strategies can differ dramatically in the case of assortative versus disassortative mixing, see (Galeotti and Rogers 2013) for a study in a population composed of two groups. This question is in particular addressed in Sect. 4 for an elementary model with an arbitrary number of groups.
(iii) **How to handle individuals with the same level of connection**. Targeting individuals that are the most connected is a common approach used to prevent an epidemic in a complex network (Pastor-Satorras and Vespignani 2002). In [17], we show that these strategies are optimal for the so-called monotonic kernel models, in which the individuals may be naturally ordered by a score related to their connectivity. When many individuals or groups are tied for the best score, either from the beginning or after some vaccine has been distributed, the optimal way of vaccinating them may be surprisingly varied according to the situation. This variety of answers appears already in the treatment of such individuals in the assortative/disassortative toy model developed in Sect. 4. To go further in this direction, a large part of the current paper, see Sects. 5–7, is devoted to regular or “constant degree” models where all individuals share the same degree. We shall in particular ask whether uniform vaccination strategies are either the “best” or the “worst” or even neither the “best” nor the “worst” possible strategies.

In most of the examples below, the next-generation matrices are symmetric. Although the optimization problems (2) and (3) make sense without symmetry assumptions (Delmas et al. 2021b), symmetry, or at least symmetrizability, is required for the convexity and concavity properties of the effective reproduction number $R_e$ proved in Delmas et al. (2021a). Note that real world data provides in general symmetric or symmetrizable contact matrices; see for example the POLYMOD matrix in Mossong et al. (2008) and the theoretical model in Busenberg and Castillo-Chavez (1991).

### Main results

Section 2 is dedicated to classical finite-dimensional metapopulation models. We present two simple models that, despite being seemingly very similar, display totally different behaviors: the asymmetric and symmetric circle graphs. For the first one, where individuals of the group *i* can only be infected by individuals of the group $i-1$ and which corresponds to a next generation matrix given by:

$$\begin{aligned} K_{ij}=\mathbb {1}_{\{i=j+1 \mod N\}}, \end{aligned}$$

with *N* the number of groups or nodes in the circle, we derive a greedy parametrization of the Pareto frontier. On the second one, where individuals of the group *i* can only be infected by individuals of the group $i-1$ or $i+1$ and which corresponds to a next generation matrix given by:

$$\begin{aligned} K_{ij}=\mathbb {1}_{\{i=j \pm 1 \mod N\}}, \end{aligned}$$

we observe numerically that the Pareto frontier is much more complicated, and in particular cannot be parametrized greedily. Those two models are in fact constant degree models; the uniform vaccination strategies are the “worst” for the first model, and neither the “best” nor the “worst” strategies for the second.

After Sect. 3, where we recall the kernel setting used in Delmas et al. (2021b) for infinite dimensional models, we focus in Sect. 4 on the effect of assortativity on optimal vaccination strategies. We define a simple kernel model that may be assortative or disassortative depending on the sign of a parameter. In the discrete metapopulation model, the next generation matrix can be written (up to a multiplicative constant) as:

$$\begin{aligned} K_{ij}= \left( 1 + \varepsilon \mathbb {1}_{\{i\ne j\}}\right) \, \mu _j , \end{aligned}$$

where $\mu _j\ge 0$ represents the proportional size of group *j*. The model is assortative if $\varepsilon <0$ (and $\varepsilon \ge -1$ so that the matrix *K* is non-negative) and disassortative if $\varepsilon >0$. We describe completely the optimal vaccination strategies, see Theorem 4.2, and show that the best strategies for the assortative case are the worst ones if the mixing pattern is disassortative, and vice-versa. We also prove that all the Pareto and anti-Pareto frontiers admit greedy parametrizations, and that Pareto optimal strategies prioritize individuals that in some sense have the highest degree, that is, are the most connected.

In Sect. 5, we consider constant degree models, which are the analogue of regular graphs in the infinite-dimensional setting. In the discrete metapopulation model, the sums over each row and the sums over each column of the next generation matrix are constant. We prove, see Proposition 5.4, that if the effective reproduction function $R_e$ is convex then the uniform strategies are the “best” and they give a greedy parametrization of the Pareto frontier; and that if $R_e$ is concave, the uniform strategies are the “worst”. Section 6 is then devoted to a particular model of rank two, which corresponds in the discrete metapopulation model to a next generation matrix of the form:

$$\begin{aligned} K_{ij}= (1+ \varepsilon \alpha _i \alpha _j)\, \mu _j \quad \text {with} \quad \sum _j \alpha _j \, \mu _j=0, \end{aligned}$$

where $$\varepsilon $$ may be $$+1 $$ or $$-1$$, and $$\sup _i \alpha _i^2\le 1$$, so that the matrix *K* is non-negative. The condition $$\sum _j \alpha _j\mu _j=0$$ ensures that the model has a constant degree. In those cases, we give a complete description of the “best” and the “worst” vaccination strategies, the uniform one being “best” for $$\varepsilon =+1$$ and “worst” otherwise, see Proposition 6.2. In Sect. 6.5, we also provide an example of kernel (in infinite dimension) for which the set of optimal strategies has an infinite number of connected components. In this particular case, there is no greedy parametrization of the Pareto frontier.

As another application of the results of Sect. 5, we investigate in Sect. 7 geometric constant degree kernels defined on the unit sphere ${\mathbb {S}^{d-1}}\subset \mathbb {R}^d$. Intuitively an individual at point *x* on the sphere is infected by an individual at point *y* with an intensity $\textrm{k}(x,y)$ depending on the distance between *x* and *y*. Those kernels appear in the graphon theory as limit of large dense random geometric graphs. We give a particular attention to the affine model in Sect. 7.3, where:

$$\begin{aligned} \textrm{k}(x,y)=1+ \varepsilon \langle x,y \rangle , \qquad \varepsilon \ge -1, \end{aligned}$$

where $\langle x,y \rangle$ is the usual scalar product in the ambient space $\mathbb {R}^d$. Intuitively, for $\varepsilon >0$, the infection propagates through the nearest neighbors: this may be seen as a kind of spatial assortativity. By contrast, for $\varepsilon <0$ the infection propagates through the furthest individuals neighbors, in a spatially disassortative way. For this affine model, we completely describe the “best” and the “worst” vaccination strategies, see Proposition 7.9.

## First examples in the discrete setting

In this section, we use the framework developped by Hill and Longini in Hill and Longini (2003) for metapopulation models and provide optimal vaccination strategies for two very simple examples. Despite their simplicity, these examples showcase a number of interesting behaviors, that will occur a in much more general setting, as we will see in the rest of the paper.

### The reproduction number in metapopulation models

In metapopulation models, the population is divided into $N \ge 2$ different subpopulations and we suppose that individuals within a same subpopulation share the same characteristics. The different groups are labeled 1, 2, ..., *N*. We denote by $\mu _1$, $\mu _2$, ..., $\mu _{N}$ their respective size (in proportion with respect to the total size) and we suppose that those do not change over time. By the linearization of the dynamic of the epidemic at the disease-free equilibrium, we obtain the so-called *next-generation matrix* *K*, see Van Den Driessche and Watmough (2002), which is a $N \times N$ matrix with non-negative coefficients. For a detailled discussion on the biological interpretation of the coefficients of the next-generation matrix, we refer the reader to (Delmas et al. 2022b, Section 2). We also refer to [14] for an extensive treatment of the two-dimensional case.

The basic reproduction number is equal to the spectral radius of the next-generation matrix:

4 $$\begin{aligned} R_0 = \rho (K), \end{aligned}$$

where $\rho$ denotes the spectral radius. Since the matrix *K* has non-negatives entries, the Perron-Frobenius theory implies that $R_0$ is also an eigenvalue of *K*. If $R_0 > 1$, the epidemic process grows away from zero infectives while if $R_0 < 1$, the disease cannot invade the population; see (Van Den Driessche and Watmough 2002 ,Theorem 2) .

We now introduce the effect of vaccination. Suppose that we have at our disposal a vaccine with perfect efficacy, *i.e.*, vaccinated individuals are completely immunized to the infection. We denote by $\eta =(\eta _1, \ldots , \eta _{N})$ the vector of the proportions of **non-vaccinated** individuals in the different groups. We shall call $\eta$ a vaccination strategy and denote by $\Delta =[0, 1]^N$ the set of all possible vaccination strategies. According to Delmas et al. (2022b, 2021b), the next-generation matrix corresponding to the dynamic with vaccination is equal to the matrix *K* multiplied by the matrix $\textrm{Diag}(\eta )$ on the right, where $\textrm{Diag}(\eta )$ is the $N \times N$ diagonal matrix with coefficients $\eta \in \Delta$. We call the spectral radius of this matrix the *effective reproduction number*:

5 $$\begin{aligned} R_e(\eta ) = \rho \left( K \cdot \textrm{Diag}(\eta ) \right) . \end{aligned}$$

The effective reproduction number accounts for the vaccinated (and immunized) people in the population, as opposed to the basic reproduction number, which corresponds to a fully susceptible population. When nobody is vaccinated, that is $\eta = {\mathbb {1}}= (1,\ldots ,1)$, $\textrm{Diag}(\eta )$ is equal to the identity matrix, the next-generation matrix is unchanged and $R_e(\eta )=R_e({\mathbb {1}})=R_0$.

We suppose that the *cost* of a vaccination strategy is, up to an irrelevant multiplicative constant, equal to the total proportion of vaccinated people and is therefore given by:

6 $$\begin{aligned} C(\eta ) = \sum _{i=1}^{N} (1 - \eta _i) \mu _i = 1 - \sum _{i=1}^{N} \eta _i \mu _i, \end{aligned}$$

where $\eta =(\eta _1, \ldots , \eta _{N})\in \Delta$. We refer to (Delmas et al. 2021b, Section 5.1, Remark 5.2) for considerations on more general cost functions.

#### Example 2.1

*(Uniform vaccination)* The uniform strategy of cost *c* consists in vaccinating the same proportion of people in each group: $\eta = (1-c){\mathbb {1}}$. By homogeneity of the spectral radius, the reproduction number $R_e(\eta )$ is then equal to $(1 - c) R_0$.

### Optimal allocation of vaccine doses

As mentioned in the introduction and recalled in Sect. 2.1, reducing the reproduction number is fundamental in order to control and possibly eradicate the epidemic. However, the vaccine may only be available in a limited quantity, and/or the decision maker may wish to limit the cost of the vaccination policy. This motivates our interest in the following related problem:

7 $$\begin{aligned} \left\{ \begin{array}{cc} \min \, &{} R_e(\eta ), \\ \text {such that} &{} C(\eta ) = c. \end{array} \right. \end{aligned}$$

According to Delmas et al. (2021b), one can replace the constraint $\{C(\eta )=c\}$ by $\{C(\eta )\le c\}$ without modifying the solutions. The opposite problem consists in finding out the *worst possible way* of allocating vaccine. While this does not seem at first sight to be as important, a good understanding of bad vaccination strategies may also provide rules of thumb in terms of anti-patterns. In order to estimate how bad a vaccination strategy can be, we therefore also consider the following problem:

8 $$\begin{aligned} \left\{ \begin{array}{cc} \max \, &{} R_e(\eta ), \\ \text {such that} &{} C(\eta ) = c. \end{array} \right. \end{aligned}$$

According to Delmas et al. (2021b), one can replace the constraint  $\{C(\eta )=c\}$ by $\{C(\eta )\ge c\}$ without modifying the solutions.

Since the coefficients of the matrix $K\cdot \textrm{Diag}(\eta )$ depend continuously on $\eta$, it is classical that its eigenvalues also depend continuously on $\eta$ (see for example Horn and Johnson 2013, Appendix D) and in particular the function $R_e$ is continuous on $\Delta =[0,1]^N$. Since the function *C* is also continuous on $\Delta$, the compactness of $\Delta$ ensures the existence of solutions for Problems (7) and (8). For  $c \in [0,1]$, $R_{e\star }(c)$ (resp. $R_e^\star (c)$) stands for the minimal (resp. maximal) value taken by $R_e$ on the set of all vaccination strategies $\eta$ such that $C(\eta ) = c$:

9 $$\begin{aligned} R_{e\star }(c)&= \min \{ R_e(\eta )\,:\, \eta \in \Delta \text { and } C(\eta ) = c \}, \end{aligned}$$

10 $$\begin{aligned} R_e^\star (c)&= \max \{ R_e(\eta )\,:\, \eta \in \Delta \text { and } C(\eta ) = c \}. \end{aligned}$$

It is easy to check that the functions $R_{e\star }$ and $R_e^\star$ are non increasing. Indeed, if $\eta ^1$ and $\eta ^2$ are two vaccination strategies such that $\eta ^1 \le \eta ^2$ (where $\le$ stands for the pointwise order), then $R_e(\eta ^1) \le R_e(\eta ^2)$ according to the Perron-Frobenius theory. This easily implies that $R_{e\star }$ and $R_e^\star$ are non-increasing. We refer to Delmas et al. (2021b, 2022b) for more properties on those functions; in particular they are also continuous. For the vaccination strategy $\eta = {\mathbb {0}}= (0,...,0)$ (everybody is vaccinated) with cost $C({\mathbb {0}}) = 1$, the transmission of the disease in the population is completely stopped, *i.e.*, the reproduction number is equal to 0. In the examples below, we will see that for some next-generation matrices *K*, this may be achieved with a strategy $\eta$ with cost $C(\eta ) < 1$. Hence, let us denote by $c_\star$ the minimal cost required to completely stop the transmission of the disease:

11 $$\begin{aligned} c_\star = \inf \{ c \in [0,1] \, :\, R_{e\star }(c) = 0 \} = \inf \{ C(\eta )\, :\, R_e(\eta )=0\}. \end{aligned}$$

In a similar fashion, we define by symmetry the maximal cost of totally inefficient vaccination strategies:

12 $$\begin{aligned} c^\star = \sup \{ c \in [0,1] \, :\, R_e^\star (c) = R_0 \}. = \sup \{ C(\eta )\, :\, R_e(\eta )=R_0\}. \end{aligned}$$

According to (Delmas et al. 2021b, Lemma 5.13(ii)), we have $c^\star = 0$ if the matrix *K* is irreducible, *i.e.*, not similar via a permutation to a block upper triangular matrix. The two matrices considered below in this section are irreducible.

Following Delmas et al. (2021b), the *Pareto frontier* associated to the “best” vaccination strategies, solution to Problem (7), is defined by:

13 $$\begin{aligned} \mathcal {F}= \{(c, R_{e\star }(c)) \, :\, c \in [0,c_\star ]\}. \end{aligned}$$

The set of “best” vaccination strategies, called *Pareto optimal* strategies, is defined by:

14 $$\begin{aligned} \mathcal {P}=\{\eta \in \Delta \, :\, (C(\eta ), R_e(\eta ))\in \mathcal {F}\}. \end{aligned}$$

When $c^\star =0$ (which will be the case for all the examples considered in this paper), the *anti-Pareto frontier* associated to the “worst” vaccination strategies, solution to Problem (8), is defined by:

15 $$\begin{aligned} \mathcal {F}^\textrm{Anti}= \{(c, R_e^\star (c)) \, :\, c \in [0, 1]\}. \end{aligned}$$

The set of “worst” vaccination strategies, called *anti-Pareto optimal* strategies, is defined by:

16 $$\begin{aligned} \mathcal {P}^\textrm{Anti}=\{\eta \in \Delta \, :\, (C(\eta ), R_e(\eta ))\in \mathcal {F}^\textrm{Anti}\}. \end{aligned}$$

The set of uniform strategies will play a role in the sequel:

17 $$\begin{aligned} \mathcal {S}^\textrm{uni}=\{ t{\mathbb {1}}\, :\, t \in [0,1]\}. \end{aligned}$$

We denote by ${\textbf{F}}=\{(C(\eta ), R_e(\eta ))\, :\, \eta \in \Delta \}$ the set of all possible outcomes. According to (Delmas et al. 2021b, Section 6.1), the set ${\textbf{F}}$ is a subset of $[0,1] \times [0,R_0]$ delimited below by the graph of $R_{e\star }$ and above by the graph of $R_e^\star$; it is compact, path connected and its complement is connected in $\mathbb {R}^2$.

A *path* of vaccination strategies is a measurable function $\gamma \, :\, [a,b] \rightarrow \Delta$ where $a<b$. It is *monotone* if for all $a \le s \le t \le b$ we have $\gamma (s) \ge \gamma (t)$, where $\le$ denotes the pointwise order. A *greedy parametrization* of the Pareto (resp. anti-Pareto) frontier is a monotone continuous path $\gamma$ such that the image of $(C \circ \gamma , R_e \circ \gamma )$ is equal to $\mathcal {F}$ (resp. $\mathcal {F}^\textrm{Anti}$). If such a path exists, then its image can be browsed by a greedy algorithm which performs infinitesimal locally optimal steps.

#### Remark 2.2

Let *K* be the next-generation matrix and let $\lambda \in \mathbb {R}_+ \backslash \{0\}$. By homogeneity of the spectral radius, we have  $\rho (\lambda K\cdot \textrm{Diag}(\eta )) = \lambda \rho (K\cdot \textrm{Diag}(\eta ))$. Thus, the solutions of Problems (7) and (8) and the value of $c_\star$ are invariant by scaling of the matrix *K*. As for the functions $R_{e\star }$ and $R_e^\star$, they are scaled by the same quantity. Hence, in our study, the value of $R_0$ will not matter. Our main concern will be to find the best and the worst vaccination strategies for a given cost and compare them to the uniform strategy.

### The fully asymmetric circle model

**Fig. 1:**
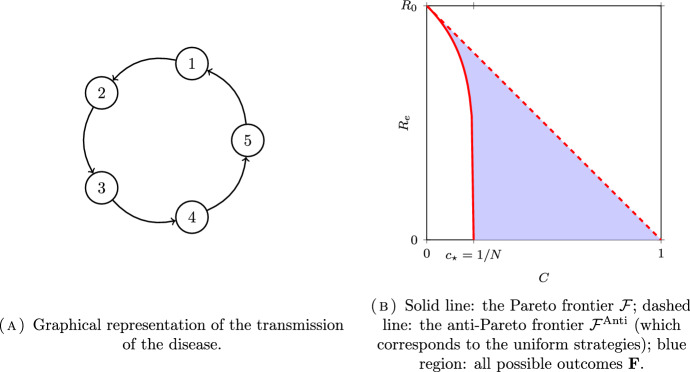
Example of optimization for the fully asymmetric circle model with $N=5$ subpopulations

We consider a model of $N\ge 2$ equal subpopulations (*i.e.* $\mu _1 = \cdots =\mu _{N} = 1/N$) where each subpopulation only contaminates the next one. The next-generation matrix, which is equal to the cyclic permutation matrix, and the effective next generation matrix are given by:

18 $$\begin{aligned} K = \begin{pmatrix} 0 &{} 1 &{} &{} &{} \\ &{} 0 &{} 1 &{} &{} \\ &{} &{} \ddots &{} \ddots &{} \\ 0 &{} &{} &{} 0 &{} 1\\ 1 &{} 0 &{} &{} &{} 0 \end{pmatrix} \quad \text {and}\quad K \cdot \textrm{Diag}(\eta ) = \begin{pmatrix} 0 &{} \eta _2 &{} &{} &{} \\ &{} 0 &{} \eta _3 &{} &{} \\ &{} &{} \ddots &{} \ddots &{} \\ 0 &{} &{} &{} 0 &{} \eta _{N} \\ \eta _{1} &{} 0 &{} &{} &{} 0 \end{pmatrix}, \end{aligned}$$

where $\eta =(\eta _1, \ldots , \eta _{N})\in \Delta =[0, 1]^N$. The next-generation matrix can be interpreted as the adjacency matrix of the fully asymmetric cyclic graph; see Fig. 1A.

By an elementary computation, the characteristic polynomial of the matrix $K \cdot \textrm{Diag}(\eta )$ is equal to $X^N - \prod _{1 \le i \le N} \eta _i$. Hence, the effective reproduction number can be computed via an explicit formula; it corresponds to the geometric mean:

19 $$\begin{aligned} R_e(\eta )= \left( \prod _{i=1}^{N} \eta _i\right) ^{1/N}. \end{aligned}$$

The Pareto and anti-Pareto frontier are totally explicit for this elementary example, and given by the following proposition. For additional comments on this example; see also Example 5.9 below.

#### Proposition 2.3

(Asymmetric circle) For the fully asymmetric circle model, we have:

(i) The least quantity of vaccine necessary to completely stop the propagation of the disease is $c_\star =1/N$. Pareto optimal strategies have a cost smaller than $c_\star$, and correspond to giving all the available vaccine to one subpopulation:
  $$\begin{aligned} \mathcal {P}=\left\{ \eta =(\eta _1, \ldots , \eta _{N})\in [0, 1]^N\, :\eta _i=1 \text { for all~ }i\text { but at most one}\right\} . \end{aligned}$$
  The Pareto frontier is given by the graph of the function $R_{e\star }$ on $[0, c_\star ]$, where $R_{e\star }$ is given by:
  $$\begin{aligned} R_{e\star }(c) = (1-Nc )_+^{1/N} \quad \text {for}\quad c\in [0,1]. \end{aligned}$$
(ii) The maximal cost of totally inefficient vaccination strategies is $c^\star =0$. The anti-Pareto optimal strategies consist in vaccinating uniformly the population, *i.e.*:
  $$\begin{aligned} \mathcal {P}^\textrm{Anti}=\mathcal {S}^\textrm{uni}. \end{aligned}$$
  The anti-Pareto frontier is given by the graph of the function $R_e^\star : c \mapsto 1-c$ on [0, 1].

In Fig. 1B, we have plotted the Pareto and the anti-Pareto frontiers corresponding to asymmetric circle model with $N=5$ subpopulations.

#### Remark 2.4

*(Greedy parametrization)* From Proposition 2.3, we see that there exists a greedy parametrization of the Pareto frontier, which consists in giving all the available vaccine to one subpopulation until its complete immunization. Similarly, the anti-Pareto frontier is greedily parametrized by the uniform strategies.

#### Proof

We first prove (i). Suppose that $c\ge 1/N$. There is enough vaccine to protect entirely one of the groups and obtain $R_e(\eta ) = 0$ thanks to Equation (19). This gives $c_\star \le 1/N$ and $R_{e\star }(c)=0$ for $c\ge 1/N$.

Let $0 \le c < 1/N$. According to (Boyd and Vandenberghe 2004, Section 3.1.5), the map $\eta \mapsto R_e(\eta )$ is concave. According to Bauer’s maximum principle (Niculescu and Persson 2006, Corollary A.3.3), $R_e$ attains its minimum on $\{ \eta \in [0, 1]^N \, :\, \, C(\eta ) = c\}$ at some extreme point of this set. These extreme points are strategies $\eta \in [0, 1]^N$ such that $\eta _i = 1 - Nc$ for some *i* and $\eta _j = 1$ for all $j \ne i$. Since $R_e$ is a symmetric function of its *N* variables, it takes the same value $(1-Nc)^{1/N}$ on all these strategies, so they are all minimizing, which proves Point (i).

We give another elementary proof of (i) when $c < 1/N$. Let $\eta$ be a solution of Problem (7). Assume without loss of generality that $\eta _1 \le \cdots \le \eta _{N}$. Suppose for a moment that $\eta _2 < 1$, and let $\varepsilon > 0$ be small enough to ensure $\eta _1>\varepsilon$ and $\eta _2 < 1 - \varepsilon$. Then the vaccination strategy ${\tilde{\eta }} = (\eta _1 - \varepsilon , \eta _2 + \varepsilon , \eta _3, \ldots ,\eta _{N})$ is admissible, and:

$$\begin{aligned} R_e({\tilde{\eta }})^N = R_e(\eta )^N - (\varepsilon (\eta _2 - \eta _1) + \varepsilon ^2) \prod _{i = 3}^{N} \eta _i< R_e(\eta )^N, \end{aligned}$$

contradicting the optimality of $\eta$. Therefore the Pareto-optimal strategies have only one term different from 1, and must be equal to $((1-Nc),1,\ldots , 1)$, up to a permutation of the indices.

Now, let us prove (ii). Let $\eta$ be such that $C(\eta ) = c$. According to the inequality of arithmetic and geometric means:

$$\begin{aligned} R_e(\eta ) \le \frac{\eta _1 + \cdots + \eta _{N}}{N} = 1-c. \end{aligned}$$

By Example 2.1, the right hand side is equal to the effective reproduction number of the uniform vaccination at cost *c*. This ends the proof of the proposition. $\square$

### Fully symmetric circle model

We now consider the case where each of the *N* subpopulation may infect both of their neighbours. The next-generation matrix and the effective next-generation matrix are given by:

20 $$\begin{aligned} K = \begin{pmatrix} 0 &{} 1 &{} &{} 0 &{} 1 \\ 1 &{} 0 &{} 1 &{} &{} 0 \\ &{} 1 &{} \ddots &{} \ddots &{} \\ 0 &{} &{} \ddots &{} 0 &{} 1\\ 1 &{} 0 &{} &{} 1 &{} 0 \end{pmatrix} \quad \text {and} \quad K\cdot \textrm{Diag}(\eta ) = \begin{pmatrix} 0 &{} \eta _2 &{} &{} 0 &{} \eta _{N} \\ \eta _1 &{} 0 &{} \eta _3 &{} &{} 0 \\ &{} \eta _2 &{} \ddots &{} \ddots &{} \\ 0 &{} &{} \ddots &{} 0 &{} \eta _{N} \\ \eta _{1} &{} 0 &{} &{} \eta _{N-1} &{} 0 \end{pmatrix} . \end{aligned}$$

**Fig. 2:**
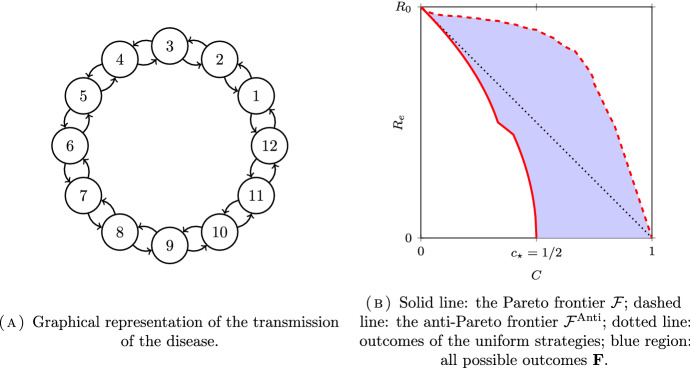
Example of optimization for the fully symmetric circle model with $N=12$ subpopulations

Again, we can represent this model as a graph; see Fig. 2A.

There is no closed-form formula to express $R_e$ for $N \ge 5$ and the optimization is much harder than the asymmetric case. Since *K* is irreducible, we have $c^\star =0$. Our only analytical result for this model is the computation of $c_\star$.

#### Proposition 2.5

(Optimal strategy for stopping the transmission) For the fully symmetric circle model, the strategy $\eta ' = \mathbb {1}_{i \, \text {even}}$ is Pareto optimal for the fully symmetric circle and $R_e(\eta ')=0$. In particular, $c_\star$ is equal to $C(\eta ')=\left\lceil \,N/2\,\right\rceil /N$.

#### Proof

The term $X^{N-2}$ of the characteristic polynomial of $K\cdot \textrm{Diag}(\eta )$ has a coefficient equal to the sum of all principal minors of size 2:

21 $$\begin{aligned} - (\eta _1 \eta _2 + \eta _2 \eta _3 + \ldots + \eta _{N-1} \eta _{N} + \eta _{N} \eta _1). \end{aligned}$$

If $\eta$ is such that $N C(\eta ) < \left\lceil \,N/2\,\right\rceil$, then at least one of the term above is not equal to 0, proving that the sum is negative. Hence, there is at least one eigenvalue of $K \cdot \textrm{Diag}(\eta )$ different from 0, and $R_e(\eta ) > 0$. We deduce that $c_\star \ge \left\lceil \,N/2\,\right\rceil /N$.

Now, let $\eta '$ be such that $\eta _i' = 0$ for all odd *i* and $\eta _i' = 1$ for all even *i*, so that $C(\eta ') = \left\lceil \,N/2\,\right\rceil /N$. The matrix $K\cdot \textrm{Diag}(\eta ')$ is nilpotent as its square is 0. Since the spectral radius of a nilpotent matrix is equal 0, we get $R_e(\eta ') = 0$. This ends the proof of the proposition.

We can give another proof of the proposition: it is enough to notice that the nodes labelled with an odd number form a maximal independent set of the cyclic graph. Taking $\eta '$ equal to the indicator function of this set, we deduce from (Delmas et al. Delmas et al. (2022b), Section 4.2) that $\eta '$ is Pareto optimal, $R_e(\eta ')=0$ and $c_\star =C(\eta ')$. $\square$

**Fig. 3:**
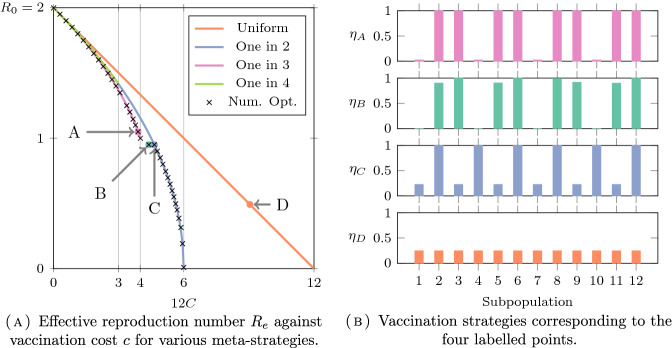
Pareto frontier and computation of the outcomes for the paths of the four meta-strategies. Some meta-strategies $\{ \eta _A, \eta _B, \eta _C, \eta _D \}$ are represented on the right with their corresponding outcome points **A**–**D** on the left

We pursue the analysis of this model with numerical computations. We choose $N=12$ subpopulations, and compute an approximate Pareto frontier, using the Borg multiobjective evolutionary algorithm.^1^ The results are plotted in Fig. 3. We represent additionnally the curves $(c, R(\eta (c)))$ where the vaccination strategy $\eta (c)$ for a given cost *c* are given by deterministic path of “meta-strategies”:

- **Uniform strategy:** distribute the vaccine uniformly to all *N* subpopulations;
- **“One in** *j*” **strategy:** vaccinate one in *j* subpopulation, for $j=2,3,4$.

Let us follow the scatter plot of $R_{e\star }$ in Fig. 3A, starting from the upper left.

1. In the beginning nobody is vaccinated, and $R_0$ is equal to 2.
2. For small costs all strategies have similar efficiency. Zooming in shows that the (numerically) optimal strateges split the available vaccine equally between four subpopulations that are separed from each other by two subpopulations. This corresponds to the “one in 3” meta-strategies path. As represented in Fig. 3B, $\eta _A$ with outcome point $A = (C(\eta _A), R_e(\eta _A))$ belongs to this path. In particular, note that disconnecting the graph is not Pareto optimal for $12c = 3$ as the disconnecting “one in 4” strategy gives values $R_e = \sqrt{2} \simeq 1.41$ opposed to the value $R_e \simeq 1.37$ for the “one in 3” strategy with same cost. However, note that, in agreement with (Delmas et al. 2022b, Proposition 5.3), this disconnecting “one in 4” strategy is also not anti-Pareto optimal, since it performs better than the uniform strategy with the same cost.
3. When $12c = 4$ the circle has been split in four “islands” of two interacting subpopulations. There is a small interval of values of *c* for which it is (numerically) optimal to split the additional vaccine uniformly between the four “islands”, and give it entirely to one subpopulation in each island: see point B and the associated strategy $\eta _B$.
4. Afterwards (see point C), it is in fact better to try and vaccinate all the (say) even numbered subpopulations. Therefore, the optimal vaccinations *do not vary monotonously* with respect to the amount of available vaccine; in other words, distributing vaccine in a greedy way is not optimal. This also suggests that, even though the frontier is continuous (in the objective space (*c*, *r*)), the set of optimal *strategies* may not be connected: the “one in two” vaccination strategy of point C cannot be linked to “no vaccination” strategy by a continuous path of optimal strategies. In particular, the Pareto frontier cannot be greedily parametrized. The disconnectedness of the set of optimal strategies will be established rigorously in Sect. 6 for another model.
5. For $12c = 6$, that is $c = c_\star$ as stated in Proposition 2.5, it is possible to vaccinate completely all the (say) odd numbered subpopulations, thereby disconnecting the graph completely. The infection cannot spread at all.
6. Even though the problem is symmetric and all subpopulations play the same role, the proportional allocation of vaccine is far from optimal; on the contrary, the optimal allocations focus on some subpopulations.

Using the same numerical algorithm, we have also computed the anti-Pareto frontier for this model; see the dashed line in Fig. 2B. Although we do not give a formal proof, the anti-Pareto frontier seems to be perfectly given by the following greedy parametrization:

1. Distribute all the available vaccine supply to one group until it is completely immunized.
2. Once this group is fully vaccinated, distribute the vaccine doses to one of its neighbour.
3. Continue this procedure by vaccinating the neighbour of the last group that has been immunized.
4. When there are only two groups left, split the vaccine equally between these two.

## The kernel model

In order to get a finer description of the heterogeneity, we could divide the population into a growing number of subgroups $N \rightarrow \infty$. The recent advances in graph limits theory (Backhausz and Szegedy 2020; Lovász 2012) justify describing the transmission of the disease by a kernel defined on a probability space. We already used this type of model in Delmas et al. (2021a, 2021b, 2022a, 2022b), in particular for an SIS dynamics, see also (Delmas et al. 2022b, Section 2) for other epidemic models.

Let $(\Omega , \mathscr {F}, \mu )$ be a probability space that represents the population: the individuals have features labeled by $\Omega$ and the infinitesimal size of the population with feature *x* is given by $\mu ({\textrm{d}}x)$. Let $L^2(\mu )$ ($L^2$ for short) be the space of real-valued measurable functions *f* defined on $\Omega$ such that $\left\Vert \,f\,\right\Vert _2=(\int _\Omega f^2\, {\textrm{d}} \mu )^{1/2}$ is finite, where functions which agree $\mu$-a.s. are identified. Let $L^2_+=\{f\in L^2\, :\, f\ge 0\}$ be the subset of non-negative functions of $L^2$. We define a *kernel* on $\Omega$ as a $\mathbb {R}_+$-valued measurable function defined on $(\Omega ^2, \mathscr {F}^{\otimes 2})$. We will only consider kernels with finite double-norm on $L^2$:22To a kernel $\textrm{k}$ with finite double-norm on $L^2$, we associate the integral operator $T_\textrm{k}$ on $L^2$ defined by:

23 $$\begin{aligned} T_\textrm{k}(g) (x) = \int _\Omega \textrm{k}(x,y) g(y)\,\mu ({\textrm{d}}y) \quad \text {for } g\in L^2 \text { and } x\in \Omega . \end{aligned}$$

The operator $T_\textrm{k}$ is bounded, and its operator norm $\left\Vert \,T_\textrm{k}\,\right\Vert _{L^2}$ satisfies:

24 $$\begin{aligned} \left\Vert \, T_\textrm{k}\,\right\Vert _{L^2} \le \left\Vert \,\textrm{k}\,\right\Vert _{2,2}. \end{aligned}$$

According to (Conway 1990, Proposition II.4.7), the operator $T_\textrm{k}$ is actually compact. A kernel is said to be symmetric if $\textrm{k}(x,y) = \textrm{k}(y,x)$, $\mu ({\textrm{d}}x) \mu ({\textrm{d}}y)$-almost surely. It is said to be *irreducible* if for all $A \in \mathscr {F}$, we have:

25 $$\begin{aligned} \int _{A \times A^c} \textrm{k}(x,y) \, \mu (\textrm{d}x) \mu (\textrm{d}y) =0 \implies \mu (A) \in \{ 0,1 \}. \end{aligned}$$

If $\textrm{k}$ is not irreducible, it is called *reducible*.

By analogy with the discrete setting and also based on Delmas et al. (2022a, 2022b), we define the basic reproduction number in this context thanks to the following formula:

26 $$\begin{aligned} R_0 = \rho (T_\textrm{k}), \end{aligned}$$

where $\rho$ stands for the spectral radius of an operator. According to the Krein-Rutman theorem, $R_0$ is an eigenvalue of $T_\textrm{k}$. Besides, there exists left and right eigenvectors associated to this eigenvalue in $L^2_+$; such functions are called Perron eigenfunctions.

For *f*, *g* two non-negative bounded measurable functions defined on $\Omega$ and $\textrm{k}$ a kernel on $\Omega$ with finite double-norm on $L^2$, we denote by $f\textrm{k}g$ the kernel on $\Omega$ defined by:

27 $$\begin{aligned} (f\textrm{k}g)(x,y) = f(x)\, \textrm{k}(x,y) g(y). \end{aligned}$$

Since *f* and *g* are bounded, the kernel $f \textrm{k}g$ has also a finite double-norm on $L^2$.

Denote by $\Delta$ the set of measurable functions defined on $\Omega$ taking values in [0, 1]. A function $\eta$ in $\Delta$ represents a vaccination strategy: $\eta (x)$ represents the proportion of **non-vaccinated** individuals with feature *x*. In particular $\eta ={\mathbb {1}}$ (the constant function equal to 1) corresponds to the absence of vaccination and $\eta ={\mathbb {0}}$ (the constant function equal to 0) corresponds to the whole population being vaccinated. The uniform strategies are given by:

$$\begin{aligned} \eta ^\textrm{uni}=t {\mathbb {1}}\quad \end{aligned}$$

for some $t\in [0, 1]$, and we denote by $\mathcal {S}^\textrm{uni}=\{ t{\mathbb {1}}\, :\, t\in [0,1]\}$ the set of uniform strategies.

The (uniform) cost of the vaccination strategy $\eta \in \Delta$ is given by the total proportion of vaccinated people, that is:

28 $$\begin{aligned} C(\eta ) = \int _\Omega (1 - \eta ) \, \textrm{d}\mu = 1- \int _\Omega \eta \, \textrm{d}\mu . \end{aligned}$$

The measure $\eta \, \textrm{d} \mu$ corresponds to the *effective population*, that is the individuals who effectively play a role in the dynamic of the epidemic. The effective reproduction number is defined by:

29 $$\begin{aligned} R_e(\eta ) = \rho (T_{\textrm{k}\eta }), \end{aligned}$$

We consider the weak topology on $\Delta$ given by the trace of the weak topology on $L^2$, so that with a slight abuse of notation we identify $\Delta$ with $\{\eta \in L^2\, :\, 0\le \eta \le 1\}$. According to Theorem 4.2 and Remark 3.2 in Delmas et al. (2021b), the function $R_e: \eta \mapsto R_e(\eta )$ is continuous on $\Delta$ with respect to the weak topology. The compactness of $\Delta$ for this topology implies the existence of solutions for Problems (7) and (8). We will conserve the same notation and definitions as in the discrete setting for: the value functions $R_{e\star }$ and $R_e^\star$, the minimal/maximal costs $c_\star$ and $c^\star$, the various sets of strategies $\mathcal {P}$ and $\mathcal {P}^\textrm{Anti}$, and the various frontiers $\mathcal {F}$ and $\mathcal {F}^\textrm{Anti}$; see Eqs. (9)–(17) in Sect. 2.2.

We shall also use the following result from (Delmas et al. 2022b, Proposition 5.1) (recall that a vaccination strategy is defined up the a.s. equality).

### Lemma 3.1

Let $\textrm{k}$ be a kernel on $\Omega$ with finite double-norm on $L^2$ such that a.s. $\textrm{k}>0$. Then, we have $c^\star =0$, $c_\star =1$ and the strategy ${\mathbb {1}}$ (resp. ) is the only Pareto optimal as well as the only anti-Pareto optimal strategy with cost $c=0$ (resp. $c=1$).

### Example 3.2

*(Discrete and continuous representations of a metapopulation model)* We recall the natural correspondence between metapopulation models (discrete models) and kernel models (continuous models) from (Delmas et al. 2021b, Section 7.4.1). Consider a metapopulation model with *N* groups given by a finite set $\Omega _{\textrm{d}} = \{ 1, 2, \ldots , N \}$ equipped with a probability measure $\mu _{\textrm{d}}$ giving the relative size of each group and a next generation matrix $K=(K_{ij}, \, i,j\in \Omega _{\textrm{d}})$. The corresponding discrete kernel $\textrm{k}_{\textrm{d}}$ on $\Omega _{\textrm{d}}$ is defined by:

30 $$\begin{aligned} K_{ij} = \textrm{k}_{\textrm{d}}(i,j) \mu _j \quad \text {where}\quad \mu _i = \mu _{\textrm{d}}(\{i\}). \end{aligned}$$

Then, the matrix $K\cdot \textrm{Diag}(\eta )$ is the matrix representation of the endomorphism $T_{\textrm{k}_{\textrm{d}} \eta }$ in the canonical basis of $\mathbb {R}^N$.

Following Delmas et al. (2021b), we can also consider a continuous representation on the state space $\Omega _{\textrm{c}} = [0,1)$ equipped with the Lebesgue measure $\mu _{\textrm{c}}$. Let $I_1 = [0, \mu _1)$, $I_2 = [\mu _1, \mu _1 + \mu _2)$, ..., $I_{N} = [1 - \mu _{N}, 1)$, so that the intervals $(I_n,\, 1 \le n \le N)$ form a partition of $\Omega$. Now define the kernel:

31 $$\begin{aligned} \textrm{k}_{\textrm{c}}(x,y) = \sum _{1\le i,j \le N} \textrm{k}_{\textrm{d}}(i,j) \mathbb {1}_{I_i \times I_j}(x,y). \end{aligned}$$

Denote by $R_e^\textrm{d}$ and $R_e^\textrm{c}$ the effective reproduction number in the discrete and continuous representation models. In the same manner, the uniform cost in each model is denoted by $C^\textrm{d}$ and $C^\textrm{c}$. According to Delmas et al. (2021b), these functions are linked through the following relation:

$$\begin{aligned} R_e^\textrm{d}(\eta ^\textrm{d}) = R_e^\textrm{c}\left( \eta ^\textrm{c} \right) , \quad \text {and} \quad C^\textrm{d}(\eta ^\textrm{d}) = C^\textrm{c}(\eta ^\textrm{c}), \end{aligned}$$

for all $\eta ^\textrm{d} \, :\, \Omega _\textrm{d} \rightarrow [0,1]$ and $\eta ^\textrm{c} \, :\, \Omega _\textrm{c} \rightarrow [0,1]$ such that:

$$\begin{aligned} \eta ^\textrm{d}(i) = \frac{1}{\mu _i} \int _{I_i} \eta ^\textrm{c}\, \textrm{d}\mu _\textrm{c} \quad \text {for all}\quad i \in \Omega _{\textrm{d}} . \end{aligned}$$

Let us recall that the Pareto and anti-Pareto frontiers for the two models are the same.

**Fig. 4:**
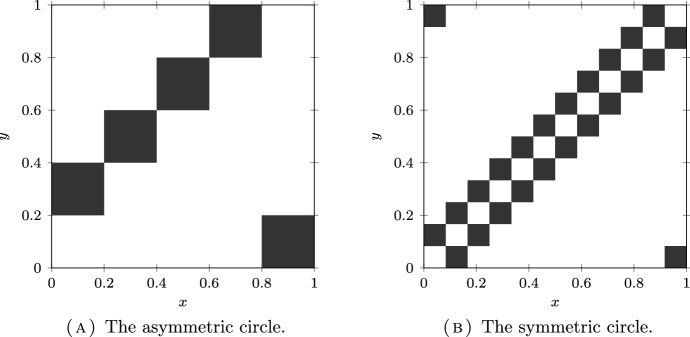
Kernels $\textrm{k}_\textrm{c}$ (equal to 0 in the white zone and to 1 in the black zone) on $\Omega _\textrm{c}=[0, 1)$ and $\mu _\textrm{c}$ the Lebesgue measure of the continuous model associated to discrete metapopulation models

In Fig. 4, we have plotted the kernels of the continuous models associated to the asymmetric and symmetric circles models from Sects. 2.3 and 2.4.

## Assortative versus disassortative mixing

### Motivation

We consider a population divided into an at most countable number of groups. Individuals within the same group interact with intensity *a* and individuals in different groups interact with intensity *b*. Hence, the model is entirely determined by the coefficients *a* and *b* and the size of the different groups. This simple model allows to study the effect of assortativity, that is, the tendency for individuals to connect with individuals belonging to their own subgroup. The mixing pattern is called *assortative* (higher interaction in the same subgroup) if $a>b$, and *disassortative* (lower interaction in the same subgroup) when $b > a$. Our results illustrate how different the optimal vaccination strategies can be between assortative and disassortative models, an effect that was previously studied by Galeotti and Rogers (2013) in a population composed of two groups.

When the population is equally split in a finite number of subgroups, and *a* is equal to 0, the next-generation matrix of this model corresponds, up to a multiplicative constant, to the adjacency matrix of a complete multipartite graph. Recall that an *m*-partite graph is a graph that can be colored with *m* different colors, so that all edges have their two endpoints colored differently. When $m = 2$ these are the so-called bipartite graphs. A complete multipartite graph is a *m*-partite graph (for some $m\in \mathbb {N}^*$) in which there is an edge between every pair of vertices from different colors.

The complete multipartite graphs have interesting spectral properties. Indeed, Smith (1970) showed that a graph with at least one edge has its spectral radius as its only positive eigenvalue if and only if its non-isolated vertices induce a complete multipartite graph. In Esser and Harary (1980), Esser and Harary proved that two complete *m*-partite graphs with the same number of nodes are isomorphic if and only if they have the same spectral radius. More precisely, they obtained a comparison of the spectral radii of two complete *m*-partite graphs by comparing the sizes of the sets in their partitions through majorization; see (Esser and Harary 1980, Lemma 3).

The goal of this section is to generalize and complete these results and give a full picture of the Pareto and anti-Pareto frontiers for the assortative and the disassortative models.

### Spectrum and convexity

We will use an integer intervals notation to represent the considered kernels. For $i, j \in \mathbb {N}\cup \{ + \infty \}$, we set $[\![i,j]\!]$ (resp. $[\![i, j[\![$) for $[i, j]\cap (\mathbb {N}\cup \{ + \infty \})$ (resp. $[i, j) \cap \mathbb {N}$). Let $N \in [\![2, + \infty ]\!]$ and $\Omega = [\![1, N]\!]$ if *N* is finite and $\Omega = [\![1, +\infty [\![$ otherwise. The set $\Omega$ is endowed with the discrete $\sigma$-algebra $\mathscr {F}=\mathcal {P}(\Omega )$ and a probability measure $\mu$. To simplify the notations, we write $\mu _i$ for $\mu (\{ i \})$ and $f_i=f(i)$ for a function *f* defined on $\Omega$. Without loss of generality, we can suppose that $\mu _i \ge \mu _j >0$ for all $i \le j$ elements of $\Omega$. We consider the kernel $\textrm{k}$ defined for $i,j \in \Omega$ by:

32 $$\begin{aligned} \textrm{k}(i,j) = {\left\{ \begin{array}{ll} a \quad \text {if} \quad i=j, \\ b \quad \text {otherwise}, \end{array}\right. } \end{aligned}$$

where *a* and *b* are two non-negative real numbers.

If $b = 0$, then the kernel is reducible, and, thanks to (Delmas et al. 2021a, Lemma 5.3), the effective reproduction number is given by the following formula: $R_e(\eta ) = a \max _{i\in \Omega } \eta _i \,\mu _i$, for all $\eta =(\eta _i, i\in \Omega )\in \Delta$. This is sufficient to treat this case and we have $c^\star = 1 - \mu _1$.

From now on, we assume that $b > 0$. The next two results describe the spectrum of $T_\textrm{k}$ in both the assortative and disassortative case. Notice the spectrum of $T_\textrm{k}$ is real as $\textrm{k}$ is symmetric. Recall that $R_0=\rho (T_\textrm{k})$.

#### Proposition 4.1

(Convexity/concavity of $R_e$) Let $\textrm{k}$ be given by (32), with $b> 0$ and $a \ge 0$.

(i) **Assortative model.** If $a\ge b> 0$, then the operator $T_\textrm{k}$ is positive semi-definite and the function $R_e$ is convex.
(ii) **Disassortative model.** If $b\ge a\ge 0$ and $b>0$, then $R_0$ is the only positive eigenvalue of $T_\textrm{k}$, and it has multiplicity one. Furthermore, the function $R_e$ is concave.

In the following proof, we shall consider the symmetric matrix $M_n$ of size $n \times n$, with $n\in \mathbb {N}^*$, given by:

$$\begin{aligned} M_n(i,j) = {\left\{ \begin{array}{ll} a \quad \text {if} \quad i=j, \\ b \quad \text {otherwise}. \end{array}\right. } \end{aligned}$$

The matrix $M_n$ is the sum of *b* times the all-ones matrix and $a - b$ times the identity matrix. Thus, $M_n$ has two distinct eigenvalues: $nb + a$ with multiplicity 1 and $a-b$ with multiplicity $n-1$.

#### Proof

We first prove (i). For any $g\in L^2$, we have:

$$\begin{aligned} \int _{\Omega \times \Omega } g(x) \textrm{k}(x,y) g(y) \, \mu (\textrm{d}x) \mu (\textrm{d} y) = a \sum _{i \in \Omega } g_i^2 \mu _i^2 + b \sum _{i \ne j} g_i g_j \, \mu _i \mu _j \ge b \left\Vert \,g\,\right\Vert _2^2. \end{aligned}$$

This implies that $T_\textrm{k}$ is positive semi-definite. Thus, as $\textrm{k}$ is symmetric, the fonction $R_e$ is convex, thanks to (Delmas et al. 2021a, Theorem 4.10).

We now prove (iii). We give a direct proof when *N* is finite, and use an approximation procedure for $N=\infty$. We first assume that *N* is finite. For $n \le N$, let $v_n = \mathbb {1}_{[\![1, n ]\!]}$ and set $T_n = T_{v_n \textrm{k}v_n}$. The non-null eigenvalues of $T_n$ (with their multiplicity) are the eigenvalues of the matrix $M_n \cdot \textrm{Diag}_n(\mu )$, where $\textrm{Diag}_n (\mu )$ is the diagonal $n\times n$-matrix with $(\mu _1, \ldots , \mu _n)$ on the diagonal. Thanks to (Horn and Johnson 2013, Theorem 1.3.22), these are also the eigenvalues of the matrix $Q_n=\textrm{Diag}_n (\mu )^{1/2} \cdot M_n \cdot \textrm{Diag}_n(\mu )^{1/2}$. By Sylvester’s law of inertia (Horn and Johnson 2013, Theorem 4.5.8), the matrix $Q_n$ has the same signature as the symmetric matrix $M_n$. In particular, since we have supposed $a-b\le 0$, $M_n$ has only one positive eigenvalue. Thus $Q_n$ has only one positive eigenvalue: thanks to the Perron-Frobenius theory, it is its spectral radius. This concludes the proof when *N* is finite by choosing $n=N$.

If $N=\infty$, we consider the limit $n\rightarrow N$. Since:

$$\begin{aligned} \lim _{n \rightarrow \infty } \left\Vert \,\textrm{k}- v_n \textrm{k}v_n\,\right\Vert _{2,2} = 0, \end{aligned}$$

the spectrum of $T_n$ converges to the spectrum of $T_\textrm{k}$, with respect to the Hausdorff distance, and the multiplicity on the non-zero eigenvalues also converge, see (Delmas et al. 2021a, Lemma 2.4). This shows that $\rho (T_\textrm{k})$ is the only positive eigenvalue of $T_\textrm{k}$, and it has multiplicity one. Since $\textrm{k}$ is symmetric, we deduce the concavity of the function $R_e$ from (Delmas et al. 2021a, Theorem 4.10). $\square$

### Explicit description of the Pareto and anti-Pareto frontiers

For $c\in [0,1]$, we define an “*horizontal vaccination*” $\eta ^{\textrm{h}}(c)\in \Delta$ with cost *c* in the following manner. Rather than defining directly the proportion of non-vaccinated people in each class, it will be convenient to define first the resulting effective population size, which will be denoted by $\xi$. For all $\alpha \in [0,\mu _1]$, let $\xi ^\textrm{h}(\alpha )\in \Delta$ be defined by:

33 $$\begin{aligned} \xi _i^\textrm{h}(\alpha ) = \min (\alpha , \mu _i), \quad i \in \Omega . \end{aligned}$$

For all $i \in \Omega$, $\xi _i^\textrm{h}(\alpha )$ is a non-decreasing and continuous function of $\alpha$. The map $\alpha \mapsto \sum _i \xi _i^\textrm{h}(\alpha )$ is continuous and increasing from $[0,\mu _1]$ to [0, 1], so for any $c\in [0,1]$, there exists a unique $\alpha _c$ such that $\sum _i \xi _i^\textrm{h}(\alpha _c) = 1-c$. We then define the horizontal vaccination profile $\eta ^{\textrm{h}}(c)\in \Delta$ by:

34 $$\begin{aligned} \eta ^{\textrm{h}}_i(c) = \xi _i^\textrm{h}(\alpha _c)/ \mu _i, \quad i \in \Omega . \end{aligned}$$

**Fig. 5:**
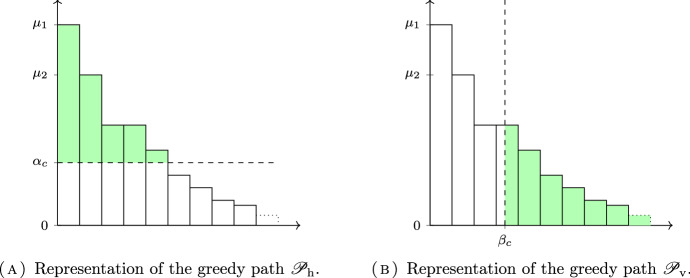
Greedy parametrization of the (anti-)Pareto front. The bar plot represents the measure $\mu$. The proportion of green in each bar correspond to the proportion of vaccinated individuals in each subpopulation

In words, it consists in vaccinating in such a way that the quantity of the non-vaccinated individuals $\xi _i^\textrm{h} = \eta _i\mu _i$ in each subpopulation is always less than the “horizontal” threshold $\alpha$: see Fig. 5A. The cost of the vaccination strategy $\eta ^{\textrm{h}}(c)$ is indeed *c*. Note that $\eta ^{\textrm{h}}(0) = {\mathbb {1}}$ (no vaccination), whereas $\eta ^{\textrm{h}}(1) = {\mathbb {0}}$ (full vaccination), and that the path $c \mapsto \eta ^{\textrm{h}}(c)$ is greedy. We denote its range by $\mathscr {P}_{\textrm{h}}$.

For $c\in [0,1]$, we define similarly a “*vertical vaccination*” $\eta ^{\textrm{v}}(c)\in \Delta$ with cost *c*. First let us define for $\beta \in [0,N]$:

35 $$\begin{aligned} \xi ^{\textrm{v}}_i(\beta ) = \mu _i \cdot \min (1, (\beta +1 - i)_+), \quad i \in \Omega . \end{aligned}$$

The map $\beta \mapsto \sum _i \xi ^{\textrm{v}}_i(\beta )$ is increasing and continuous from [0, *N*] to [0, 1], so for any $c\in [0,1]$ there exists a unique $\beta _c$ such that $\sum _i \xi ^{\textrm{v}}_i(\beta _c) = 1-c$. We then define the vertical vaccine profile $\eta ^{\textrm{v}}(c)$ by:

36 $$\begin{aligned} \eta ^{\textrm{v}}_i(c) = \xi ^{\textrm{v}}_i(\beta _c)/\mu _i, \quad i \in \Omega . \end{aligned}$$

In words, if $\lceil \beta \rceil = \ell$, this consists in vaccinating all subpopulations *j* for $j>\ell$, and a fraction $\lceil \beta \rceil -\beta$ of the subpopulation $\ell$, see Fig. 5B for a graphical representation. The cost of the vaccination strategy $\eta ^{\textrm{v}}(c)$ is by construction equal to *c*.

For all $i \in \Omega$, $\eta ^{\textrm{v}}_i(c)$ is a non-increasing and continuous function of *c*. Just as in the horizontal case, we have $\eta ^{\textrm{v}}(0)={\mathbb {1}}$ (no vaccination), $\eta ^{\textrm{v}}(1)={\mathbb {0}}$ (full vaccination), and the path $c \mapsto \eta ^{\textrm{v}}(\beta (c))$ is also greedy. We denote its range by $\mathscr {P}_{\textrm{v}}$.

These two paths give a greedy parametrization of the Pareto and anti-Pareto frontiers for the assortative and disassortative models: more explicitly, we have the following result, whose proof can be found in Sect. 4.4.

#### Theorem 4.2

(Assortative vs disassortative) Let $\textrm{k}$ be given by (32), with $b> 0$ and $a \ge 0$.

(i) *Assortative model.* If $a\ge b>0$, then $\mathscr {P}_{\textrm{v}}$ and $\mathscr {P}_{\textrm{h}}$ are greedy parametrizations of the anti-Pareto and Pareto frontiers respectively.
(ii) *Disassortative model.* If $b\ge a>0$, then $\mathscr {P}_{\textrm{v}}$ and $\mathscr {P}_{\textrm{h}}$ are greedy parametrizations of the Pareto and anti-Pareto frontiers respectively.
(iii) *Complete multipartite model.* If $a =0$ and $b > 0$, then $\mathscr {P}_{\textrm{h}}$ is a greedy parametrization of the anti-Pareto frontier and the subset of strategies $\eta \in \mathscr {P}_{\textrm{v}}$ such that $C(\eta ) \le 1 - \mu _0$ is a greedy parametrization of the Pareto frontier. In particular, we have $c_\star = 1 - \mu _1$ and $c^\star =0$.

Notice that $c^\star =0$ and $c_\star =1$ in cases (i) and (ii) as $\textrm{k}$ is positive thanks to Lemma 3.1.

#### Remark 4.3

*(Highest Degree vaccination)* The effective degree function of a symmetric kernel $\textrm{k}$ at $\eta \in \Delta$ is the function $\textsf{deg}_\eta$ defined on $\Omega$ by:

37 $$\begin{aligned} \textsf{deg}_\eta (x) = \int _\Omega \textrm{k}(x,y) \eta (y) \, \mu (\textrm{d}y). \end{aligned}$$

When $\eta = {\mathbb {1}}$, it is simply called the degree of $\textrm{k}$ and is denoted by $\textsf{deg}$. In our model, the effective degree of the subgroup *i* is given by

38 $$\begin{aligned} \textsf{deg}_\eta (i) = a\eta _ i \mu _i + b \sum _{\ell \ne i} \eta _\ell \mu _\ell , \end{aligned}$$

and thus the degree of the subgroup *i* is given by $$\textsf{deg}(i) = (a-b) \mu _i + b$$. As $$\mu _i\ge \mu _j$$ for $$ i< j$$ elements of $$\Omega $$, we deduce that the degree function is monotone: non-increasing in the assortative model and non-decreasing in the disassortative model. The group with the highest degree therefore corresponds to the largest group in the assortative model and the smallest group (if it exists) in the disassortative model.

Consider the assortative model where all the groups have different size, *i.e.*, $\mu _1> \mu _2 > \ldots$ Following the parametrization $c\mapsto \eta ^{\textrm{h}}(c)$, starting from $c=0$, will first decrease the effective size of the group 1 (the group with the highest degree) until it reaches the effective degree of group 2 (with the second highest degree). Once these two groups share the same effective degree which corresponds to reaching $\mu _1 \eta ^{\textrm{h}}_1=\mu _2$, they are vaccinated uniformly (that is, ensuring that they keep the same effective degree: using (38) this corresponds to  $\mu _1 \eta ^{\textrm{h}}_1=\mu _2 \eta ^{\textrm{h}}_2$) until their effective degree is equal to the third highest degree, and so on and so forth.

In the disassortative model, the function $\textsf{deg}_\eta$ remains (strictly) increasing when the vaccination strategies in $\mathscr {P}_{\textrm{v}}$ are applied. In particular, if $\mu _1> \mu _2 > \ldots$, then the optimal strategies prioritize the groups with the higher effective degree until they are completely immunized. If multiple groups share the same degree, it is optimal to give all available doses to one group.

In conclusion, in both models, the optimal vaccination consists in vaccinating the groups with the highest effective degree in priority if this group is unique. But if multiple groups share the same degree (*i.e.*, have the same size), the optimal strategies differ between the assortative and the disassortative case. In the assortative case, groups with the same size must be vaccinated uniformly while in the disassortive case, all the vaccine doses shall be given to one group until it is completely vaccinated.

#### Example 4.4

*(Group sizes following a dyadic distribution)* Let $$N = \infty $$, $$\Omega =\mathbb {N}^*$$ and $$\mu _i = 2^{-i}$$ for all $$i \in \Omega $$. Following (Delmas et al. 2021b, Section 7.4.1), we will couple this discrete model with a continuum model for a better visualization on the figures. Let $$\Omega _c = [0,1)$$ be equipped with the Borel $$\sigma $$-field $$\mathscr {F}_c$$ and the Lebesgue measure $$\mu _c$$. The set $$\Omega _c$$ is partitionned into a countable number of intervals $$I_i = [ 1 - 2^{-i+1}, 1 - 2^{-i})$$, for $$i \in \mathbb {N}^*$$, so that $$\mu _c(I_i) = \mu _i$$. The kernel of the continuous model corresponding to $$\textrm{k}$$ in (32) is given by:

39 $$\begin{aligned} \textrm{k}_c = (a-b) \sum _{i\in \mathbb {N}^*} \mathbb {1}_{I_i \times I_i} + b {\mathbb {1}}. \end{aligned}$$

**Fig. 6:**
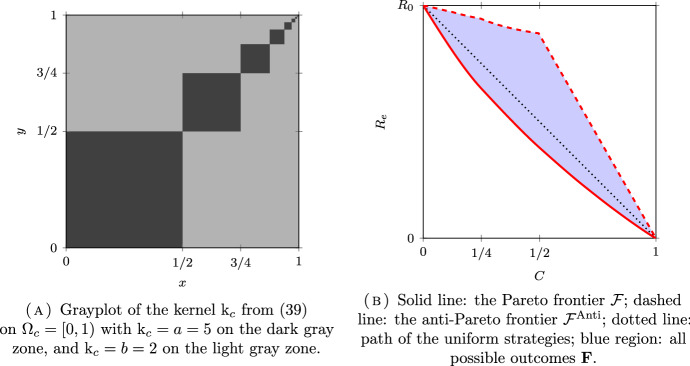
An assortative model

**Fig. 7:**
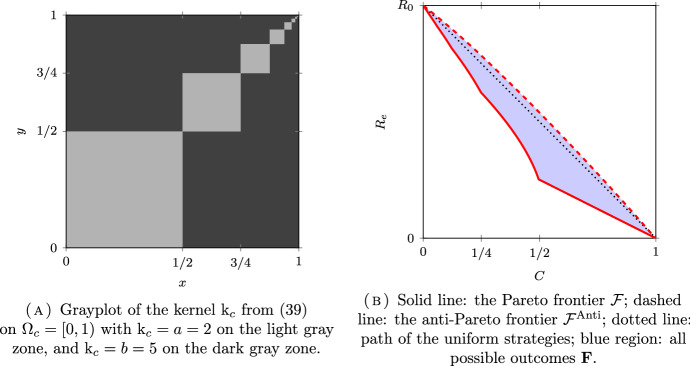
A disassortative model

**Fig. 8:**
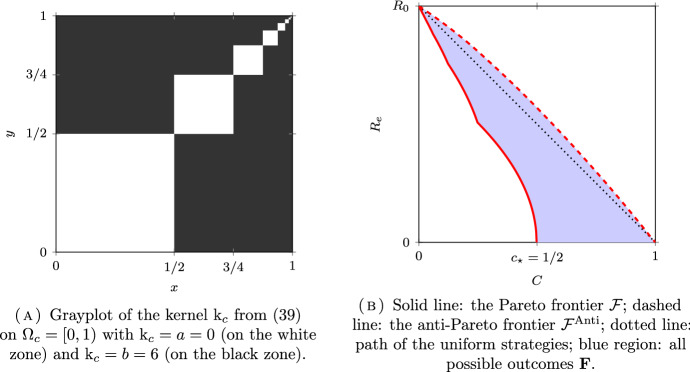
An example of the complete multipartite model

The kernel $\textrm{k}_c$ is plotted in Figs. 6A, 7A and 8A for different values of *a* and *b* corresponding respectively to the assortative, the disassortative and the complete multipartite case corresponding to points (i), (ii) and (iii) of Theorem 4.2 respectively. Their respective Pareto and anti-Pareto frontiers are plotted in Figs. 6B, 7B and 8B, using a finite-dimensional approximation of the kernel $\textrm{k}$ and the power iteration method. In Fig. 8B, the value of $c_\star$ is equal to $1-\mu _1=1/2$. With this continuous representation of the population, the set $\mathscr {P}_{\textrm{v}}$ corresponds to the strategies of the form $\mathbb {1}_{[0, t)}$ for $t \in [0,1]$.

Notice that the Pareto frontier in the assortative case is convex. This is consistent with (Delmas et al. 2021b, Proposition 6.6) since the cost function is affine and $R_e$ is convex when $a \ge b$; see Proposition 4.1 (i). In the same manner, the anti-Pareto frontier in the disassortative and the multipartite cases is concave. Once again, this is consistent with (Delmas et al. 2021b, Proposition 6.6) since the cost function is affine and $R_e$ is concave when $b \ge a$; see Proposition 4.1 (iii).

### Proof of Theorem 4.2

After recalling known facts of majorization theory, we first consider the finite dimension models, and then the general case by an approximation argument.

#### Majorization

In this section, we recall briefly some definitions and results from majorization theory, and refer to Arnold (1987); Marshall et al. (2011) for an extensive treatment of this topic.

Let $n \ge 1$ and $\xi ,\chi \in \mathbb {R}^{n}_+$. We denote by $\xi ^\downarrow$ and $\chi ^\downarrow$ their respective order statistics, that is the vectors in $\mathbb {R}_+^{n}$ with the same components, but sorted in descending order. We say that $\xi$ is *majorized* by $\chi$, and write $\xi \prec \chi$, if:

40 $$\begin{aligned} \sum _{j=1}^{i} \xi _j^\downarrow \le \sum _{j=1}^i \chi _j^\downarrow \quad \text {for all~}i\in \{1, \ldots , n\},\text { and}\quad \sum _{j=1}^{n} \xi _j = \sum _{j=1}^{n} \chi _j. \end{aligned}$$

Among the various characterizations of majorization, we will use the following by Hardy, Littlewood and Pólya; see (Marshall et al. 2011, Proposition I.4.B.3):

41 $$\begin{aligned} \xi \prec \chi \iff \sum _{i=1}^n (\xi _i - t)_+ \le \sum _{i=1}^n (\chi _i - t)_+ \quad \text {for all}\quad t\in \mathbb {R}_+, \end{aligned}$$

where $u_+ = \max (u, 0)$, for all $u \in \mathbb {R}$. A real-valued function $\Theta$ defined on $\mathbb {R}_+^{n}$ is called *Schur-convex* if it is non-decreasing with respect to $\prec$, that is, $\xi \prec \chi$ implies $\Theta (\xi ) \le \Theta (\chi )$. A function $\Theta$ is called *Schur-concave* if $(-\Theta )$ is Schur-convex.

#### Schur-convexity and concavity of the spectral radius in finite dimension

We define the function $\Theta _n$ on $\mathbb {R}_+^{n}$ by:

$$\begin{aligned} \Theta _n(\xi ) = \rho (M_n\cdot \textrm{Diag}(\xi )), \end{aligned}$$

where $\textrm{Diag}(\xi )$ is the diagonal $n\times n$-matrix with $\xi$ on the diagonal. By construction, for $\eta =(\eta _1, \ldots , \eta _n, 0, \ldots )$, we have:

42 $$\begin{aligned} R_e(\eta ) = \Theta _n(\eta _1 \mu _1, \ldots , \eta _n \mu _n). \end{aligned}$$

The key property below will allow us to identify the optimizers.

##### Lemma 4.5

(Schur-concavity and Schur-convexity) Let $b >0$ and $a \ge 0$. The function $\Theta _n$ is Schur-convex if $a \ge b$, and Schur-concave if $a \le b$.

##### Proof

Let us consider the disassortative case where $a \le b$. By a classical result of majorization theory (Marshall et al. 2011, Proposition I.3.C.2.), it is enough to show that $\Theta _n$ is symmetric and concave.

To prove that $\Theta _n$ is symmetric, consider $\sigma$ a permutation of $\{1, 2, \ldots , n \}$ and $P_\sigma$ the associated permutation matrix of size $n \times n$. Since $P_\sigma M_n P_\sigma ^{-1} = M_n$, we deduce that $\Theta _n(\xi _{\sigma }) = \Theta _n(\xi )$, where $\xi _\sigma$ is the $\sigma$-permutation of $\xi \in \mathbb {R}_+^{n}$. Thus $\Theta _n$ is symmetric.

We now prove that $\Theta _n$ is concave on $\mathbb {R}_+^{n}$. Since $R_e$ is concave thanks to Proposition 4.1 (iii), we deduce from (42), that the function $\Theta _n$ is concave on $[0, \mu _1] \times \ldots \times [0, \mu _n]$. Since $\Theta _n$ is homogeneous, it is actually concave on the whole domain $\mathbb {R}_+^n$. This concludes the proof when $a \le b$.

The proof is the same for the assortative case $a \ge b$, replacing the reference to Proposition 4.1 (iii) by (i). $\square$

#### Extreme vaccinations for fixed cost

Let us show that the horizontal and vertical vaccinations give extreme points for the preorder $\prec$ on finite sets, when the quantity of vaccine is fixed. Recall that $\xi ^\textrm{h}$ and $\xi ^\textrm{v}$ are defined in (33) and (35) respectively.

##### Proposition 4.6

(Extreme vaccinations) Let $n\in \Omega$, $\beta \in [0,n)$ and $\alpha \in [0, \mu _1]$. Let $\xi ^{\textrm{v},n} = (\xi ^\textrm{v}_1(\beta ), \ldots ,\xi ^{\textrm{v}}_n(\beta ))$, and $\xi ^{\textrm{h},n} = (\xi ^{\textrm{h}}_1(\alpha ), \ldots , \xi ^{\textrm{h}}_n(\alpha ))$. For any $\xi = (\xi _1,\ldots , \xi _n)\in [0,\mu _1]\times \cdots \times [0,\mu _n]$, we have:

$$\begin{aligned} \left( \sum _{i=1}^n \xi _i = \sum _{i=1}^n \xi ^{\textrm{v},n}_i \right) \implies \xi \prec \xi ^{\textrm{v},n}, \quad \text {and} \quad \left( \sum _{i=1}^n \xi _i = \sum _{i=1}^n \xi ^{\textrm{h},n}_i \right) \implies \xi ^{\textrm{h},n} \prec \xi . \end{aligned}$$

##### Proof

Let $\xi \in [0,\mu _1]\times \cdots \times [0,\mu _n]$ be such that $\sum _{i=1}^n \xi _i = \sum _{i=1}^n \xi ^{\textrm{v},n}_i$. The reordered vector $\xi ^\downarrow$ clearly satisfies the same conditions, so without loss of generality we may assume that $\xi$ is sorted in descending order. Using Equation (35), we get:

$$\begin{aligned} \sum _{i=1}^\ell \xi _i \le \sum _{i=1}^\ell \mu _i = \sum _{i=1}^\ell \xi ^{\textrm{v},n}_i, \quad \text {for} \quad 1 \le \ell \le \left\lfloor \,\beta \,\right\rfloor . \end{aligned}$$

We also have:

$$\begin{aligned} \sum _{i=1}^\ell \xi _i \le \sum _{i=1}^n \xi _i = \sum _{i=1}^n \xi ^{\textrm{v},n}_i = \sum _{i=1}^\ell \xi ^{\textrm{v},n}_i, \quad \text {for} \quad \ell > \left\lfloor \,\beta \,\right\rfloor . \end{aligned}$$

Therefore, we get $\xi \prec \xi ^{\textrm{v},n}$, by the definition of $\prec$.

Similarly, let $\xi \in [0,\mu _1]\times \cdots \times [0,\mu _n]$ be such that $\sum _{i=1}^n \xi _i = \sum _{i=1}^n \xi ^{\textrm{h},n}_i$. If $t\ge \alpha$ then:

$$\begin{aligned} \sum _i( \xi ^{\textrm{h},n}_i - t)_+ = 0 \le \sum _i (\xi _i-t)_+, \end{aligned}$$

while if $t\in [0, \alpha )$, using the fact that $\sum _{i=1}^n\xi _i = \sum _{i=1}^n\xi ^{\textrm{h},n}_i$, the expression $\xi _i^{\textrm{h},n} = \min (\alpha ,\mu _i)$, and the inequalities $\xi _i\le \mu _i$, we get:

$$\begin{aligned} \sum _{i=1}^n (\xi ^{\textrm{h},n}_i - t)_+&= \sum _{i=1}^n(\xi ^{\textrm{h},n}_i - t) + \sum _{i=1}^n (t-\xi ^{\textrm{h},n}_i)_+ \\&= \sum _{i=1}^n(\xi _i - t) + \sum _{i=1}^n (t-\mu _i)_+ \\&\le \sum _{i=1}^n(\xi _i -t) + \sum _{i=1}^n (t-\xi _i)_+ \\&= \sum _{i=1}^n (\xi _i - t)_+. \end{aligned}$$

This gives $\xi ^{\textrm{h},n} \prec \xi$, by the characterization (41). $\square$

#### “Vertical” Pareto optima in the disassortative case

We consider here the disassortative model $b\ge a\ge 0$ and $b>0$. Let $c \in (0,1)$ and $D(c)= \{ \eta \in \Delta \, :\, C(\eta ) = c \}$ be the set of vaccination strategies with cost *c*. We will solve the constrained optimization Problem (7) that corresponds to:

43 $$\begin{aligned} \left\{ \begin{array}{cc} \min \, &{} R_e(\eta ), \\ \text {such that} &{} \eta \in D(c). \end{array} \right. \end{aligned}$$

Recall the definitions of $\beta _c$ and $\eta ^{\textrm{v}}(c)$ given page 36. Let $\eta \in D(c)$. Let *n* be large enough so that $\sum _{j>n} \mu _j< 1-c$ so that $\sum _{j\le n} \eta _j \mu _j>0$, and assume that $n>\beta _c$. Let $\eta ^{(n)}\in \Delta$ be defined by:

$$\begin{aligned} \eta ^{(n)}_i = \frac{\sum _{j\le n} \eta ^{\textrm{v}}_j(c) \mu _j}{\sum _{j\le n} \eta _j \mu _j} \mathbb {1}_{\{i\le n\}} \, \eta _i. \end{aligned}$$

Note that since $C(\eta ^{\textrm{v}}(c)) = c = C(\eta )$, we have $\lim _{n \rightarrow N} \eta ^{(n)} = \eta$ (pointwise and in $L^2$). Let $\xi ^n = (\eta _1^{(n)} \mu _1, \ldots , \eta _n^{(n)} \mu _n)$ and $\xi ^{\textrm{v}, n}$ be defined as in Proposition 4.6 with $\beta = \beta _c$. By construction, we have $\sum _{i=1}^n \xi _i^n = \sum _{i=1}^n \xi _i^{\textrm{v},n}$, so by Proposition 4.6, we get $\xi ^n \prec \xi ^{\textrm{v},n}$. This implies that:

$$\begin{aligned} R_e(\eta ^{(n)} ) = \Theta _n(\xi ^n) \ge \Theta _n(\xi ^{\textrm{v},n}) = R_e(\eta ^{\textrm{v}}(c)), \end{aligned}$$

where the inequality follows from the Schur concavity of $\Theta _n$ in the disassortative case (see Lemma 4.5) and where the last equality holds as $n \ge \left\lceil \,\beta _c\,\right\rceil$. Since $R_e$ is continuous and $\eta ^{(n)}$ converges pointwise and in $L^2$ to $\eta$, we get $R_e(\eta )\ge R_e(\eta ^{\textrm{v}})$. This implies that $\eta ^{\textrm{v}}$ is a solution of Problem (43).

If $a > 0$, then $\textrm{k}$ is positive everywhere, and we deduce from Lemma 3.1 that $c_\star = 1$. If $a=0$, it is easy to prove that $\{0\}$ is a maximal independant set of $\textrm{k}$; this gives that $c_\star =1-\mu _1$, thanks to (Delmas et al. 2022b, Remark 4.5). Since for all $c\in [0,c_\star )$ there exists $\eta \in \mathscr {P}_{\textrm{v}}$ such that $C(\eta )=c$, we also get that $\mathscr {P}_{\textrm{v}}\cap \{ \eta \in \Delta \, :\, C(\eta ) \le c_\star \}$ is a parametrization of the Pareto frontier. This gives the parametrization of the Pareto frontier using $\mathscr {P}_{\textrm{v}}$ from Theorem 4.2 (ii) and (iii).

#### “Horizontal” anti-Pareto optima in the disassortative case

We still consider $b \ge a \ge 0$ and $b>0$. Let $c \in (0,1)$. We now turn to the anti-Pareto frontier by studying the constrained maximization Problem (8) that corresponds to:

44 $$\begin{aligned} \left\{ \begin{array}{cc} \max \, &{} R_e(\eta ), \\ \text {such that} &{} \eta \in D(c). \end{array} \right. \end{aligned}$$

Recall the definitions of $\alpha _c$ and $\eta ^{\textrm{h}}(c)$ given page 34. Let $\eta \in D(c)$. Let *n* be large enough so that $\sum _{j>n} \mu _j< 1-c$ and thus $\sum _{j\le n} \eta _j \mu _j>0$. Define $\eta ^{(n)}\in \Delta$ by:

$$\begin{aligned} \eta ^{(n)}_i = \frac{\sum _{j\le n} \eta ^{\textrm{h}}_j(c) \mu _j}{\sum _{j\le n} \eta _j \mu _j} \mathbb {1}_{\{i\le n\}} \, \eta _i. \end{aligned}$$

Let $\xi ^n = (\eta _1^{(n)} \mu _1, \ldots , \eta _n^{(n)} \mu _n)$ and let $\xi ^{\textrm{h}, n}$ be defined as in Proposition 4.6 with $\alpha = \alpha _c$. By construction, we have $\sum _{i=1}^n \xi _i^n = \sum _{i=1}^n \xi _i^{\textrm{h},n}$, so by Proposition 4.6, we obtain $\xi ^{\textrm{h},n} \prec \xi ^n$. This implies that:

$$\begin{aligned} R_e(\eta ^{(n)} ) = \Theta _n(\xi ^n) \le \Theta _n(\xi ^{\textrm{h},n}) = R_e(\eta ^{\textrm{h}}(c) \, \mathbb {1}_{[\![1, n ]\!]}), \end{aligned}$$

where the inequality follows from the Schur concavity of $\Theta _n$.

Now, as *n* goes to infinity $\eta ^{(n)}$ converges pointwise and in $L^2$ to $\eta$, and $\eta ^{\textrm{h}}(c) \, \mathbb {1}_{[\![1, n ]\!]}$ converges pointwise and in $L^2$ to $\eta ^{\textrm{h}}(c)$, so by continuity of $R_e$ we get $R_e(\eta ) \le R_e(\eta ^{\textrm{h}}(c))$, and $\eta ^{\textrm{h}}(c)$ is solution of the Problem (44) and is thus anti-Pareto optimal for $c\in (0, 1)$ as $c^\star =0$. Since $c^\star =0$, we also deduce from (Delmas et al. 2021b, Propsotion 5.8 (iii)) that  and ${\mathbb {1}}$ are anti-Pareto optimal. Since for all $c\in [0, 1]$ there exists $\eta \in \mathscr {P}_{\textrm{h}}$ such that $C(\eta )=c$, we deduce that $\mathscr {P}_{\textrm{h}}$ is a parametrization of the anti-Pareto frontier.

#### The assortative case

The case $a \ge b>0$, corresponding to point (i) in Proposition 4.2, is handled similarly, replacing concavity by convexity, minima by maxima and vice versa.

## Constant degree kernels and unifom vaccinations

### Motivation

We have seen in the previous section an example of model where vaccinating individuals with the highest degree is the best strategy. A similar phenomenon is studied in [17], where under monotonicity arguments on the kernel, vaccinating individuals with the highest (resp. lowest) degree is Pareto (resp. anti-Pareto) optimal. However, in case multiple individuals share the same maximal degree, the optimal strategies differ completely between the assortative and the disassortative models: the Pareto optimal strategies for one model correspond to the anti-Pareto optimal strategies for the other and vice versa.

Motivated by this curious symmetry, we investigate in this section constant degree kernels, that is, the situation where all the individuals have the same number of connections. In Sect. 5.2, we define these kernels formally and give the main result on the optimality of the uniform strategies when $R_e$ is either convex or concave, see Proposition 5.4. Section 5.3 is devoted to the proof of this main result. We study in more detail the optimal strategies in an example of constant degree symmetric kernels of rank two in Sect. 6. Eventually, we study in Sect. 7 geometric kernels on the sphere, which are constant degree kernels.

### On the uniform strategies for constant degree kernels

In graph theory, a regular graph is a graph where all vertices have the same number of in-neighbors, and the same number of out-neighbors. In other words all vertices have the same in-degree and the same out-degree. Limits of undirected regular graphs have been studied in details by Backhausz and Szegedy (2020) and Kunszenti-Kovács et al. (2021). When the graphs are dense, their limit can be represented as a regular graphon, that is a symmetric kernel with a constant degree function.

Since we do not wish to assume symmetry, we give the following general definition. For a kernel $\textrm{k}$ on $\Omega$, we set, for all $z\in \Omega$ and $A\in \mathscr {F}$:

$$\begin{aligned} \textrm{k}(z, A)=\int _A \textrm{k}(z,y)\, \mu (\textrm{d} y) \quad \text {and}\quad \textrm{k}(A,z)=\int _A \textrm{k}(x,z)\, \mu (\textrm{d} x). \end{aligned}$$

For $z\in \Omega$, its in-degree is $\textrm{k}(z, \Omega )$ and its out-degree is $\textrm{k}(\Omega , z)$.

#### Definition 5.1

*(Constant degree kernel)* A kernel $\textrm{k}$ with a finite $L^2$ double-norm and positive spectral radius $R_0>0$ is called *constant degree* if all the in-degrees and all the out-degrees have the same value, that is, the maps $x\mapsto \textrm{k}(x, \Omega )$ and $y\mapsto \textrm{k}(\Omega , y)$ defined on $\Omega$ are constant, and thus equal.

#### Remark 5.2

Let $\textrm{k}$ be a constant degree kernel with spectral radius $R_0 > 0$. Notice the condition “all the in-degrees and out-degrees have the same value” is also equivalent to ${\mathbb {1}}$ being a left and right eigenfunction of $T_\textrm{k}$. We now check that the corresponding eigenvalue is $R_0$.

Let $h \in L^2_+(\Omega ) \backslash \{ {\mathbb {0}}\}$ be a left Perron-eigenfunction. Denote by $\lambda$ the eigenvalue associated to ${\mathbb {1}}$. Then, we have:

$$\begin{aligned} \lambda \int _\Omega h(x) \, \mu (\textrm{d}x) = \int _\Omega h(x) \textrm{k}(x,y) \mu (\textrm{d}x) \mu (\textrm{d}y) = R_0 \int _\Omega h(y) \, \mu (\textrm{d}y), \end{aligned}$$

where the first equality follows from the regularity of $\textrm{k}$ and from the fact that *h* is a left Perron-eigenfunction of $T_\textrm{k}$. Since *h* is non-negative and not equal to  almost everywhere, we get that $\lambda = R_0$ and ${\mathbb {1}}$ is a right Perron-eigenvector of $T_\textrm{k}$. With a similar proof, we show that ${\mathbb {1}}$ is a left Perron-eigenvector of $T_\textrm{k}$. In particular, if $\textrm{k}$ is constant degree, then the reproduction number is given by:

45 $$\begin{aligned} R_0 = \int _{\Omega \times \Omega } \textrm{k}(x,y) \, \mu (\textrm{d}x) \mu (\textrm{d}y). \end{aligned}$$

#### Example 5.3

We now give examples of constant degree kernels.

(i) Let $G=(V, E)$ be a finite non-oriented simple graph, and $\mu$ the uniform probability measure on the vertices *V*. The degree of a vertex $x\in V$ is given by
  $$\begin{aligned} \deg (x)=\sharp \{y\in V\, :\, (x,y)\in E\}. \end{aligned}$$
  The graph *G* is constant degree if all its vertices have the same degree, say $d\ge 1$. Then the kernel defined on the finite space $\Omega =V$ by the adjacency matrix is constant degree with $R_0=d$. Notice it is also symmetric.
(ii) Let $G=(V, E)$ be a finite directed graph, and $\mu$ be the uniform probability measure on the vertices *V*. The in-degree of a vertex $x \in V$ is given by
  $$\begin{aligned} \deg _\mathrm{{in}}(x) = \sharp \{ y \in V \, :\, (y,x)\in E\}, \end{aligned}$$
  and the out-degree is given by
  $$\begin{aligned} \deg _\mathrm{{out}}(x)= \sharp \{y\in V\, :\, (x,y)\in E\}. \end{aligned}$$
  The graph *G* is regular if all its vertices have the same in-degree and out-degree, say $d\ge 1$. Then the kernel defined on the finite space $\Omega =V$ by the adjacency matrix is regular with $R_0=d$. Notice it might not be symmetric.
(iii) Let $\Omega = (\mathbb {R}/ (2 \pi \mathbb {Z}))^m$ be the *m*-dimensional torus endowed with its Borel $\sigma$-field $\mathscr {F}$ and the normalized Lebesgue measure $\mu$. Let *f* be a measurable square-integrable non-negative function defined on $\Omega$. We consider the geometric kernel on $\Omega$ defined by:
  $$\begin{aligned} \textrm{k}_f(x,y)=f(x-y). \end{aligned}$$
  The kernel $\textrm{k}_f$ has a finite double-norm as $f\in L^2$. The operator $T_{\textrm{k}_f}$ corresponds to the convolution by *f*, and its spectral radius is given by $R_0 = \int _\Omega f \, \textrm{d}\mu$. Then the kernel $\textrm{k}_f$ is constant degree as soon as *f* is not equal to 0 almost surely. This example is developed in Sect. 7 in the case $m=1$ (corresponding to $d=2$ therein), see in particular Examples 7.2 and 7.3.
(iv) More generally, let $(\Omega , \cdot )$ be a compact topological group and let $\mu$ be its left Haar probability measure. Let *f* be non-negative square-integrable function on $\Omega$. Then the kernel $\textrm{k}_f(x,y) = f(y^{-1} \cdot x)$ is constant degree.

We summarize our main result in the next proposition, whose proof is given in Sect. 5.3. We recall that a strategy is called uniform if it is constant over $\Omega$.

#### Proposition 5.4

(Uniform strategies for constant degree kernels) Let $\textrm{k}$ be a constant degree kernel on $\Omega$.

(i) If the map $R_e$ defined on $\Delta$ is convex, then all uniform strategies are Pareto optimal (*i.e.* $\mathcal {S}^\textrm{uni}\subset \mathcal {P}$). Consequently, $c_\star = 1$, the Pareto frontier is the segment joining $(0, R_0)~$ to (1, 0), and for all $c \in [0,1]$:
  $$\begin{aligned} R_{e\star }(c) = (1 - c) R_0. \end{aligned}$$
(ii) If the map $R_e$ defined on $\Delta$ is concave, then the kernel $\textrm{k}$ is irreducible and all uniform strategies are anti-Pareto optimal (*i.e.* $\mathcal {S}^\textrm{uni}\subset \mathcal {P}^\textrm{Anti}$). Consequently, $c^\star = 0$, the anti-Pareto frontier is the segment joining $(0, R_0)~$ to (1, 0), and for all $c \in [0,1]$:
  $$\begin{aligned} R_e^\star (c) = (1-c) R_0. \end{aligned}$$

In (Delmas et al. 2021a, Section 4.2), we give sufficient condition on the spectrum of $T_\textrm{k}$ to be either concave or convex. Combining this result with Proposition 5.4, we get the following corollary.

#### Corollary 5.5

Let $\textrm{k}$ be a constant degree symmetric kernel.

(i) If the eigenvalues of $T_\textrm{k}$ are non-negative, then the uniform vaccination strategies are Pareto optimal and $c_\star =1$ (*i.e.* $\mathcal {S}^\textrm{uni}\subset \mathcal {P}$).
(ii) If $R_0$ is a simple eigenvalue of $T_\textrm{k}$ and the others eigenvalues are non-positive, then the kernel *k* is irreducible, the uniform vaccination strategies are anti-Pareto optimal and $c^\star =0$ (*i.e.* $\mathcal {S}^\textrm{uni}\subset \mathcal {P}^\textrm{Anti}$).

#### Remark 5.6

*(Equivalent conditions)* Let $\textrm{k}$ be a constant degree symmetric kernel. The eigenvalues of the operator $T_\textrm{k}$ are non-negative if and only if $T_\textrm{k}$ is semi-definite positive, that is:

46 $$\begin{aligned} \int _{\Omega \times \Omega } \textrm{k}(x,y) g(x) g(y) \mu (\textrm{d}x) \mu (\textrm{d}y) \ge 0 \quad \text {for all}\quad g \in L^2. \end{aligned}$$

Similarly, the condition given in Corollary 5.5 (ii) that implies the concavity of $R_e$ is equivalent to the semi-definite negativity of $T_\textrm{k}$ on the orthogonal of ${\mathbb {1}}$:

47 $$\begin{aligned} \int _{\Omega \times \Omega } \textrm{k}(x,y) g(x) g(y) \mu (\textrm{d}x) \mu (\textrm{d}y) \le 0 \quad \text {for all}\quad g \in L^2 \quad \text {such that}\quad \int _\Omega g\, \textrm{d} \mu = 0. \end{aligned}$$

#### Remark 5.7

(Comparison with a result from Poghotanyan et al. (2018)) (Poghotanyan et al. 2018, Theorem 4.7) obtained a similar result in finite dimension using a result from Friedland (1981): if the next-generation non-negative matrix *K* of size $N \times N$ satisfies the following conditions

(i) $\sum _{j=1}^{N} K_{ij}$ does not depend on $i\in [\![1, N ]\!]$ (which corresponds the parameters $a_i$ in (Poghotanyanet al. 2018, Equation (2.4)) being all equal),
(ii) $\mu _i K_{ij} = \mu _j K_{ji}$ for all $i,j \in [\![1, N ]\!]$ where $\mu _i$ denote the relative size of population *i* (which corresponds to (Poghotanyan et al. 2018), Equation (2.4)),
(iii) *K* is not singular and its inverse is an M-matrix (*i.e.*, its non-diagonal coefficients are non-positive),

then the uniform strategies are Pareto optimal (*i.e.*, they minimize the reproduction number among all strategies with same cost). Actually, this can be seen as a direct consequence of Corollary 5.5 (i). Indeed, the corresponding kernel $\textrm{k}_\textrm{d}$ defined by (30) in the discrete probability space $\Omega = [\![1, N ]\!]$ endowed with the discrete probability measure $\mu _\textrm{d}$ also defined by (30) has constant degree thanks to Point (i) and is symmetric thanks to Point (ii). Since $K^{-1}$ is an M-matrix, its real eigenvalues are positive according to (Berman and Plemmons, 1994, Chapter 6 Theorem 2.3). The eigenvalues of  $T_{\textrm{k}_\textrm{d}}$ and *K* are actually the same as *K* is the representation matrix of $T_{\textrm{k}_\textrm{d}}$ in the canonic basis of $\mathbb {R}^N$. We conclude that the operator $T_{\textrm{k}_\textrm{d}}$ is positive definite. Hence Corollary 5.5 (i) can be applied to recover that the uniform strategies are Pareto optimal.

However, Points (i) and (ii) togeteher with the positive-definitiveness of *K* do not imply Point (iii). As a counter-example, consider a population divided in $N=3$ groups of same size (*i.e*, $\mu _1 = \mu _2 = \mu _3 = 1/3$) and the following next-generation matrix:

$$\begin{aligned} K = \begin{pmatrix} 3 &{} 2 &{} 0 \\ 2 &{} 2 &{} 1 \\ 0 &{} 1 &{} 4 \end{pmatrix} \qquad \text {with inverse} \quad K^{-1} = \begin{pmatrix} 1.4 &{} -1.6 &{} 0.4 \\ -1.6 &{} 2.4 &{} -0.6 \\ 0.4 &{} - 0.6 &{} 0.4 \end{pmatrix}. \end{aligned}$$

Clearly Points (i) and (ii) hold and Point (iii) fails as $K^{-1}$ is not an M-matrix. Nevertheless, the matrix *K* is definite positive as its eigenvalues $\sigma (K) = \{ 5, 2 + \sqrt{3}, 2 - \sqrt{3} \}$ are positive. And thus, thanks to Corollary 5.5 (i), we get that the uniform strategies are Pareto optimal. Hence, Corollary 5.5 (i) is a strict generalization of (Poghotanyan et al. 2018, Theorem 4.7) even for finite metapopulation models.

#### Remark 5.8

We also refer the reader to the paper of Friedland and Karlin (1975): from the Inequality (7.10) therein, we can obtain Corollary 5.5 (i) when $\Omega$ is a compact set of $\mathbb {R}^n$, $\mu$ is a finite measure, $\textrm{k}$ is a continuous symmetrizable kernel such that $\textrm{k}(x, x)>0$ for all $x\in \Omega$. Further comments on related results may be found in (Delmas et al. 2021a, Section 4).

Below, we give examples of metapopulation models from the previous sections where Proposition 5.4 applies. For continuous models, we refer the reader to Sections 6 and 7.

#### Example 5.9

*(Fully asymmetric cycle model)* We consider the fully asymmetric circle model with $N \ge 3$ vertices developed in Sect. 2.3. Since the in and out degree of each vertex is exactly one, the adjacency matrix is constant degree according to Example 5.3 (ii).

The spectrum of the adjency matrix is given by the *N*th roots of unity, so for $N \ge 3$ it does not lie in $\mathbb {R}_-\cup \{R_0\}$, so Corollary 5.5 does not apply. However, in this case the effective spectral radius $R_e$ is given by formula (19), which corresponds to the geometric mean. According to (Boyd and Vandenberghe 2004, Section 3.1.5), the map $\eta \mapsto R_e(\eta )$ is concave, so Proposition 5.4 (ii) applies. This proves that the spectral condition given in Corollary 5.5 and in (Delmas et al. 2021a, Section 4.1) to get the concavity of $R_e$ is only sufficient.

#### Example 5.10

*(Finite assortative and disassortative model)* Let $\Omega = \{ 1,2, \ldots , N \}$ and $\mu$ be the uniform probability on $\Omega$. Let $a, b\in \mathbb {R}_+$. We consider the kernel from the models developed in Sect. 4:

$$\begin{aligned} \textrm{k}(i,j) = a \mathbb {1}_{i=j} + b \mathbb {1}_{i \ne j}. \end{aligned}$$

Since $\mu$ is uniform, the kernel $\textrm{k}$ is constant degree; provided its spectral radius is positive, *i.e.*, *a* or *b* is positive.

In the assortative model $0<b \le a$, according to Proposition 4.1 (i), the eigenvalues of the symmetric operator $T_\textrm{k}$ are non-negative. Hence, Corollary 5.5 (i) applies: the uniform strategies are Pareto optimal. This is consistent with Theorem 4.2 (i).

In the dissortative model, we have $0 \le a \le b$ and $b > 0$. According to Proposition 4.1 (iii), the eigenvalues of $T_\textrm{k}$ different from its spectral radius are non-positive. Hence, Corollary 5.5 (ii) applies: the uniform strategies are anti-Pareto. This is consistent with Theorem 4.2 (ii) and (iii).

### Proof of Proposition 5.4

By analogy with (Eaves et al. 1985), we consider the following definition.

#### Definition 5.11

(*Completely reducible kernels*) A kernel $\textrm{k}$ is said to be *completely reducible* if there exist an at most countable index set *I*, and measurable sets $\Omega _0$ and $(\Omega _i, i\in I)$, such that $\Omega$ is the disjoint union $\Omega = \Omega _0 \sqcup (\bigsqcup _{i\in I} \Omega _i)$, the kernel $\textrm{k}$ decomposes as $\textrm{k}=\sum _{i\in I} \mathbb {1}_{\Omega _i} \textrm{k}\mathbb {1}_{\Omega _i}$ a.e., and, for all $i\in I$, the kernel $\textrm{k}$ restricted to $\Omega _i$ is irreducible with positive spectral radius.

As in the discrete case for so-called line sum symmetric matrices, see (Eaves et al. 1985, Lemma 1), kernels for which for any *x* the out-degree is equal to the in-degree are necessarily completely reducible; the fact that these degrees do not depend on *x* impose further constraints.

#### Lemma 5.12

(Complete reduction) If $\textrm{k}$ is a constant degree kernel on $\Omega$, then $\textrm{k}$ is completely reducible. Furthermore, the set $\Omega _0$ from Definition 5.11 is empty, the cardinal of the partition $(\Omega _i, i\in I)$ is equal to the multiplicity of $R_0$ and thus is finite; and, for all $i \in I$, the kernel $\textrm{k}$ restricted to $\Omega _i$ is a constant degree irreducible kernel with spectral radius equal to $R_0$.

#### Proof

We recall that a set $A\in \mathscr {F}$ is invariant if $\textrm{k}(A^c, A)=0$, where for $A, B \in \mathscr {F}$:

$$\begin{aligned} \textrm{k}(B, A)=\int _{B\times A} \textrm{k}(x, y)\, \mu (\textrm{d}x)\mu (\textrm{d}y). \end{aligned}$$

Since for each *x*, the in-degree $\textrm{k}(x,\Omega )$ is equal to the out-degree $\textrm{k}(\Omega ,x)$, we get by integration $\textrm{k}(A,\Omega ) = \textrm{k}(\Omega ,A)$, so

$$\begin{aligned} \textrm{k}(A^c,A) = \textrm{k}(A^c,\Omega ) - k(A^c,A^c) = k(\Omega ,A^c) - k(A^c,A^c) = k(A,A^c). \end{aligned}$$

Therefore if *A* is invariant, then so is its complement $A^c$. According to (Delmas et al. 2021a, Section 5) and more precisely Remark 5.1(viii), there exists then an at most countable partition of $\Omega$ made of $\Omega _0$ and $(\Omega _i, i\in I)$ such that $\textrm{k}=\sum _{i\in I} \textrm{k}_i$, with $\textrm{k}_i=\mathbb {1}_{\Omega _i}\textrm{k}\mathbb {1}_{\Omega _i}$, $\mu (\Omega _i)>0$ and $\textrm{k}_i$ restricted to $\Omega _i$ is irreducible with positive spectral radius. Since ${\mathbb {1}}$ is an eigenvector of $T_\textrm{k}$ associated to the eigenvalue $R_0$ and the sets $\Omega _0$ and $(\Omega _i, i\in I)$ are pairwise disjoint, we deduce that $\Omega _0$ is of zero measure and $\mathbb {1}_{\Omega _i}$ is an eigenvector of $T_{\textrm{k}_i}$ with eigenvalue $R_0>0$, for all $i \in I$. Hence, all the kernels $\textrm{k}_i$ restricted to $\Omega _i$ are irreducible constant degree kernels with spectral radius equal to $R_0$. Thus, the cardinal of *I* is equal to the multiplicity of $R_0$ (for $T_\textrm{k}$). Since $\textrm{k}$ has finite $L^2$ double-norm, the operator $T_\textrm{k}$ is compact, and the multiplicity of $R_0>0$, and thus the cardinal of *I*, is finite. $\square$

#### Lemma 5.13

Let $\textrm{k}$ be a constant degree irreducible kernel on $\Omega$. Then the uniform strategy is a critical point for $R_e$ among all the strategies with the same cost in (0, 1), and more precisely: for all $\eta$ with the same cost in (0, 1) as $\eta ^\textrm{uni}\in \mathcal {S}^\textrm{uni}$ and $\varepsilon >0$ small enough, we have:

$$\begin{aligned} R_e((1-\varepsilon ) \eta ^\textrm{uni}+ \varepsilon \eta )= R_e(\eta ^\textrm{uni})+O(\varepsilon ^2). \end{aligned}$$

#### Proof

Let $\eta ^\textrm{uni}$ be the uniform strategy with cost $c\in (0, 1)$. Since $\textrm{k}$ is irreducible, we get that $(1-c)R_0$ is a simple isolated eigenvalue of $\textrm{k}\eta ^\textrm{uni}$, whose corresponding left and right eigenvector are ${\mathbb {1}}$ as $\textrm{k}\eta ^\textrm{uni}$ is also constant degree. For $\eta \in \Delta$, we get that $T_{\textrm{k}((1-\varepsilon ) \eta ^\textrm{uni}+ \varepsilon \eta )}$ converges to $T_{\textrm{k}\eta ^\textrm{uni}}$ (in operator norm, thanks to (24)) as $\varepsilon$ goes down to 0. Notice that:

$$\begin{aligned} \left\Vert \, T_{\textrm{k}(\eta ^\textrm{uni}+ \varepsilon (\eta - \eta ^\textrm{uni}))} - T_{\textrm{k}\eta ^\textrm{uni}}\,\right\Vert _{L^2}^2= O(\varepsilon ^2). \end{aligned}$$

According to (Kloeckner 2019, Theorem 2.6), we get that for any $\eta \in \Delta$ and $\varepsilon >0$ small enough:

$$\begin{aligned} R_e((1-\varepsilon ) \eta ^\textrm{uni}+ \varepsilon \eta )- R_e(\eta ^\textrm{uni})&= \varepsilon \int _\Omega \textrm{k}(x,y) (\eta (y) - \eta ^\textrm{uni}(y)) \, \mu (\textrm{d}x) \mu (\textrm{d}y) + O(\varepsilon ^2) \\&= \varepsilon R_0 \int _\Omega (\eta (y) - \eta ^\textrm{uni}(y)) \,\mu (\textrm{d}y) + O(\varepsilon ^2), \end{aligned}$$

where for the last equality we used that $\textrm{k}$ is constant degree. In particular, if $\eta$ and $\eta ^\textrm{uni}$ have the same cost $c\in (0, 1)$, then $R_e((1-\varepsilon ) \eta ^\textrm{uni}+ \varepsilon \eta ) - R_e(\eta ^\textrm{uni})=O(\varepsilon ^2)$, which means that the uniform strategy is a critical point for $R_e$ among all the strategies with cost $c\in (0, 1)$. $\square$

#### Proof of Proposition 5.4

We prove (i), and thus consider $\textrm{k}$ constant degree and $R_e$ convex. We first consider the case where $\textrm{k}$ is irreducible. For any $\eta$, Lemma 5.13 and the convexity of $R_e$ imply that

$$\begin{aligned} R_e(\eta ^\textrm{uni}) + O(\varepsilon ^2) = R_e((1-\varepsilon )\eta ^\textrm{uni}+ \varepsilon \eta ) \le (1-\varepsilon )R_e(\eta ^\textrm{uni}) + \varepsilon R_e(\eta ), \end{aligned}$$

where $\eta ^\textrm{uni}$ the uniform strategy with the same cost as $\eta$. Letting $\varepsilon$ go to 0, we get $R_e(\eta )\ge R_e(\eta ^\textrm{uni})$, so $R_e$ is minimal at $\eta ^\textrm{uni}$.

Since $C(\eta ^\textrm{uni})=c$ and $R_e(\eta ^\textrm{uni})=(1-c) R_0$, we deduce that $R_{e\star }(c) = (1 - c) R_0$ and thus, the Pareto frontier is a segment given by $\mathcal {F}= \{ (c, (1-c)R_0) \, :\, c\in [0, 1] \}$.

In what follows, we write $R_e[\textrm{k}]$ to stress that the reproduction function on $\Delta$ defined by (29) depends on the kernel $\textrm{k}$: $R_e[\textrm{k}](\eta )=\rho (T_{\textrm{k}\eta })$ for $\eta \in \Delta$. If $\textrm{k}$ is not irreducible, then use the representation from Lemma 5.12 (or Delmas et al. 2021a, Lemma 5.3), to get that $R_e[\textrm{k}]=\max _{i\in I} R_e[\textrm{k}_i]$. Since the cost is affine, we deduce that a strategy $\eta$ with $R_e[\textrm{k}](\eta )=\ell \in [0, R_0]$ is Pareto optimal if and only if, for all $i\in I$, the strategies $\eta _i=\eta \mathbb {1}_{\Omega _i}$ are Pareto optimal for the kernel $\textrm{k}$ restricted to $\Omega _i$ and $R_e[\textrm{k}_i](\eta _i)=\ell$; see also (Delmas et al. 2022b, Proposition 5.7). Then the first step of the proof yields that $\eta _i = \ell \mathbb {1}_{\Omega _i}$ and thus the uniform strategy $\eta ^\textrm{uni}=\ell \mathbb {1}_{\Omega }$ is Pareto optimal. This ends the proof of (i).

We now prove  (ii). We first check that the kernel $\textrm{k}$ is irreducible. Thanks to Lemma 5.12, the kernel $\textrm{k}$ is completely reducible with a zero measure $\Omega _0$. However, (Delmas et al. 2021a, Lemma 5.10) also implies that it is *monatomic*, a notion introduced in (Delmas et al. 2021a, Section 5.2) which intuitively states that $\textrm{k}$ has only one irreducible component. Together with complete reducibility, this implies that $\textrm{k}$ is irreducible. The rest of the proof is then similar to the proof of (i) under the irreducibility assumption. $\square$

## Constant degree symmetric kernels of rank two

### Motivation

Consider the integral operator $T_k$ on $L^2$ associated to a kernel *k* with finite double norm on $L^2$. According to (Conway 1990, p. 267), the operator $T_k$ is an Hilbert-Schmidt integral operator. If furthermore the kernel *k* is symmetric, thanks to the spectral theorem for compact operators (Conway 1990, Theorem II.7.6), we have the following decomposition in $L^2(\Omega ^2, \mu ^{\otimes 2})$:

$$\begin{aligned} k(x,y) = \sum _{0 \le n < N} \varepsilon _n \alpha _n(x)\alpha _n(y), \end{aligned}$$

where $0 \le N \le +\infty$, $\varepsilon _n\in \{-, +\}$ and $(\alpha _n, \, 0 \le n < N)$ is an orthogonal family of eigenvectors of $T_k$ such that $\varepsilon _n \left\Vert \,\alpha _n\,\right\Vert ^2$ is equal to the eigenvalue associated to $\alpha _n$. In particular, for constant degree symmetric kernel *k* and assuming that the rank of $T_k$ is at least two ($N\ge 2$), $\alpha _0$ is equal to ${\mathbb {1}}$ and the decomposition writes:

$$\begin{aligned} k(x,y) = R_0+ \sum _{1 \le n < N} \varepsilon _n \alpha _n(x)\alpha _n(y), \end{aligned}$$

where $\alpha _n$ for $1 \le n < N$ is orthogonal to ${\mathbb {1}}$. The integral operator associated to the kernel $R_0 + \varepsilon _1 \alpha _1(x) \alpha _1(y)$ is the best $\left\Vert \,\cdot \,\right\Vert _{L^2}$-approximation of $T_k$ by an operator of rank two if $\left\Vert \,\alpha _1\,\right\Vert \ge \left\Vert \,\alpha _n\,\right\Vert$ for all $1 \le n < N$.

Because it completes the study of the previous section but also because it can give some insights on the shape of the Pareto and anti-Pareto frontier for general symmetric constant degree kernels according to the stability results (Delmas et al. 2021b, Proposition 4.3 and Porposition 6.2), we will treat the case of symmetric constant degree kernels whose associated operator is of rank two, where one can explicitely minimize and maximize $R_e$ among all strategies at a given cost.

### Pareto and anti-Pareto frontiers

We suppose that $\Omega = [0,1)$ is equipped with the Borel $\sigma$-field $\mathscr {F}$ and a probability measure $\mu$ whose cumulative distribution function $\varphi$, defined by $\varphi (x)=\mu ([0,x])$ for $x\in \Omega$, is continuous and increasing. We consider the following two kernels on $\Omega$:

48 $$\begin{aligned} \textrm{k}^\varepsilon (x,y) = R_0 + \varepsilon \alpha (x) \alpha (y), \quad \text {with}\quad \varepsilon \in \{-, +\}, \end{aligned}$$

where $R_0 > 0$ and $\alpha \in L^2$ is strictly increasing and satisfies:

49 $$\begin{aligned} \sup _{\Omega } \alpha ^2 \le R_0 \quad \text {and}\quad \int _\Omega \alpha \,\textrm{d} \mu = 0. \end{aligned}$$

#### Remark 6.1

*(Generality)* We note that this particular choice of $\Omega$ may be made without loss of generality, and that the strict monotonicity assumption on $\alpha$ is almost general: we refer the interested reader to Sect. 6.3 for further discussion on this point.

For $\varepsilon \in \{-, +\}$, the kernel $\textrm{k}^\varepsilon$ is symmetric and constant degree. Furthermore, we have that $R_0$ and $\varepsilon \int _\Omega \alpha ^2 \,\textrm{d} \mu$ are the only non-zero eigenvalues (and their multiplicity is one) of $T_{\textrm{k}^\varepsilon }$ with corresponding eigen-vector ${\mathbb {1}}$ and $\alpha$. Since $\alpha ^2 \le R_0$, we also get that $R_0$ is indeed the spectral radius of $T_{\textrm{k}^\varepsilon }$.

The Pareto (resp. anti-Pareto) frontier is already greedily parametrized by the uniform strategies for the kernel $\textrm{k}^+$ (resp. $\textrm{k}^-$), see Corollary 5.5. The following result restricts the choice of anti-Pareto (resp. Pareto) optimal strategies to two extreme strategies. Hence, in order to find the optima, it is enough to compute and compare the two values of $R_e$ for each cost.

We recall the set of uniform strategies $\mathcal {S}^\textrm{uni}=\{ t{\mathbb {1}}\, :\, t\in [0,1]\}$ and consider the following sets of extremal strategies:

$$\begin{aligned} \mathcal {S}_0= \left\{ \mathbb {1}_{[0, t)}\, :\, t \in [0,1]\right\} \quad \text {and}\quad \mathcal {S}_1= \left\{ \mathbb {1}_{[t,1)}\, :\, t \in [0, 1]\right\} \end{aligned}$$

as well as the following set of strategies which contains $\mathcal {S}^\textrm{uni}$ thanks to (49):

$$\begin{aligned} \mathcal {S}^{\bot \alpha }=\left\{ \eta \in \Delta \, :\, \int _\Omega \alpha \, \eta \, \textrm{d} \mu =0\right\} . \end{aligned}$$

Recall that strategies are defined up to the a.s. equality. The proof of the next proposition is given is Sect. 6.4

#### Proposition 6.2

(Optima are uniform or on the sides) Let [0, 1) be endowed with a probability measure whose cumulative distribution function is increasing and continuous. Let $\textrm{k}^\varepsilon$ be given by (48) with $R_0>0$ and $\alpha$ a strictly increasing function on [0, 1) such that (49) holds.

(i) **The kernel** $\textrm{k}^+$. A strategy is Pareto optimal if and only if it belongs to $\mathcal {S}^{\bot \alpha }$. In particular, for any $c\in [0,1]$, the strategy $(1-c){\mathbb {1}}$ costs *c* and is Pareto optimal. The only possible anti-Pareto strategies of cost *c* are $\mathbb {1}_{[0, 1-c)}$ and $\mathbb {1}_{[c, 1)}$. In other words,
  $$\begin{aligned} \mathcal {P}=\mathcal {S}^{\bot \alpha }\quad \text {and}\quad \mathcal {P}^\textrm{Anti}\subset \mathcal {S}_0\cup \mathcal {S}_1. \end{aligned}$$
(ii) **The kernel** $\textrm{k}^-$. A strategy is anti-Pareto optimal if and only if it belongs to $\mathcal {S}^{\bot \alpha }$. In particular, for any $c\in [0,1]$, the strategy $(1-c){\mathbb {1}}$ costs *c* and is anti-Pareto optimal. The only possible Pareto strategies of cost *c* are $\mathbb {1}_{[0, 1-c)}$ and $\mathbb {1}_{[c, 1)}$. In other words,
  $$\begin{aligned} \mathcal {P}\subset \mathcal {S}_0\cup \mathcal {S}_1\quad \text {and}\quad \mathcal {P}^\textrm{Anti}=\mathcal {S}^{\bot \alpha }. \end{aligned}$$

In both cases, we have $c^\star =0$ and $c_\star =1$.

#### Remark 6.3

Intuitively, the populations $\{\alpha <0\}$ and $\{\alpha >0\}$ behave in an assortative way for $\textrm{k}^+$ and in a disassortative way for $\textrm{k}^-$. As in Sect. 4, the uniform strategies are Pareto optimal in the “assortative” $\textrm{k}^+$ case and anti-Pareto optimal in the “disassortative” $\textrm{k}^-$ case.

#### Remark 6.4

**Fig. 9:**
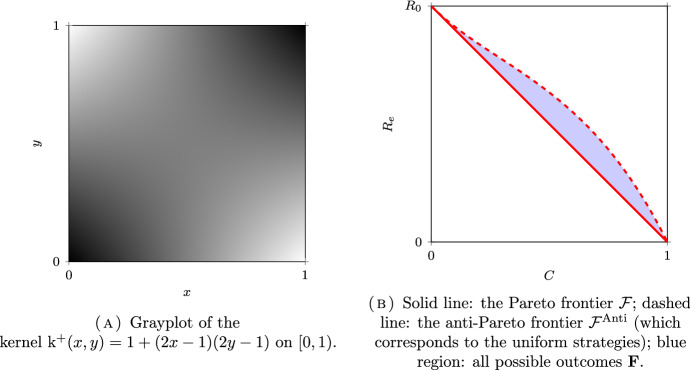
An example of a constant degree kernel operator of rank two

Under the assumptions of Proposition 6.2, if furthermore $\alpha$ is anti-symmetric with respect to 1/2, that is $\alpha (x)=-\alpha (1-x)$ for $x\in (0, 1)$, and $\mu$ is symmetric with respect to 1/2, that is $\mu ([0, x])=\mu ([1-x,1))$, then it is easy to check from the proof of Proposition 6.2 that the strategies from $\mathcal {S}_0$ and $\mathcal {S}_1$ are both optimal: $\mathcal {P}^\textrm{Anti}= \mathcal {S}_0\cup \mathcal {S}_1$ for $\textrm{k}^+$ and $\mathcal {P}= \mathcal {S}_0\cup \mathcal {S}_1$ for $\textrm{k}^-$. We plotted such an instance of $\textrm{k}^+$ and the corresponding Pareto and anti-Pareto frontiers in Fig. 9. We refer to Sect. 6.5 for an instance where $\alpha$ is not symmetric and $\mathcal {P}\ne \mathcal {S}_0\cup \mathcal {S}_1$ for $\textrm{k}^-$.

### On the choice of $\Omega =[0, 1)$ and on the monotonicity assumption

Using a reduction model technique from (Delmas et al. 2021b, Section 7), let us first see that there is no loss of generality by considering the kernel $\textrm{k}^\varepsilon =R_0 + \varepsilon \alpha \otimes \alpha$ on $\Omega =[0, 1)$ endowed with the Lebesgue measure $\mu$ and with $\alpha$ non-decreasing.

Suppose that the function $\alpha$ in (48) is replaced by an $\mathbb {R}$-valued measurable function $\alpha _0$ defined on a general probability space $(\Omega _0, \mathscr {F}_0, \mu _0)$ such that (49) holds. Thus, with obvious notations, for $\varepsilon \in \{-, +\}$, the kernel $R_0+\varepsilon \alpha _0\otimes \alpha _0$ is a kernel on $\Omega _0$. Denote by *F* the repartition function of $\alpha _0$ (that is, $F(r)=\mu _0(\alpha _0\le r)$ for $r\in \mathbb {R}$) and take $\alpha$ as the quantile function of $\alpha _0$, that is, the right continuous inverse of *F*. Notice the function $\alpha$ is defined on the probability space $(\Omega , \mathscr {F}, \mu )$ is non-decreasing and satisfies (49). Consider the probability kernel $\kappa \, :\, \Omega _0 \times \mathscr {F}\rightarrow [0,1]$ defined by $\kappa (x, \cdot )=\delta _{F(\alpha _0(x))}(\cdot )$, with $\delta$ the Dirac mass, if $\alpha$ is continuous at $\alpha _0(x)$ (that is, $F(\alpha _0(x)-)=F(\alpha _0(x))$) and the uniform probability measure on $[F(\alpha _0(x)-), F(\alpha _0(x))]$ otherwise. On the measurable space $(\Omega _0 \times \Omega , \mathscr {F}_0 \otimes \mathscr {F})$, we consider the probability measure $\nu (\textrm{d}x_1,\textrm{d}x_2) = \mu _0(\textrm{d}x_1) \kappa (x_1, \textrm{d}x_2)$, whose marginals are exactly $\mu _0$ and $\mu$. Then, for $\varepsilon \in \{-, +\}$, we have that :

$$\begin{aligned} R_0 +\varepsilon \alpha _0(x_1) \alpha _0(y_1) = R_0 + \varepsilon \alpha (x_2) \alpha (y_2) \quad \nu (\textrm{d}x_1, \textrm{d}x_2) \otimes \nu (\textrm{d}y_1, \textrm{d}y_2)\text {-a.s.} \end{aligned}$$

According to (Delmas et al. 2021b, Section 7.3), see in particular Proposition 7.3 therein, the kernels $R_0+\varepsilon \alpha _0\otimes \alpha _0$ and $R_0+\varepsilon \alpha \otimes \alpha$ are coupled and there is a correspondence between the corresponding (anti-)Pareto optimal strategies and their (anti-)Pareto frontiers are the same.

Hence, there is no loss in generality in assuming that the function $\alpha$ in (48) is indeed defined on [0, 1) and is non-decreasing.

On the contrary, one cannot assume in full generality that $\alpha$ is *strictly* increasing, as when it is only non-decreasing, the situation is more complicated. Indeed, let us take the parameters $R_0 = 1$ and $\alpha = \mathbb {1}_{[0,0.5)} - \mathbb {1}_{[0.5,1)}$. Then, the kernel $\textrm{k}^-$ is complete bi-partite: $\textrm{k}^- = \mathbb {1}_{[0,0.5) \times [0.5,1)} + \mathbb {1}_{[0.5,1) \times [0,0.5)}$. Hence, according to Theorem 4.2 (iii), we have $c_\star = 0.5$ for the kernel $\textrm{k}^-$. In a similar fashion, one can see that $\textrm{k}^+ = \mathbb {1}_{[0,0.5) \times [0,0.5)} + \mathbb {1}_{[0.5,1) \times [0.5,1)}$ is assortative and reducible; it is then easy to check that $c^\star = 0.5$ for the kernel $\textrm{k}^+$. However, it is still true that, for all costs *c*:

-  $\mathbb {1}_{[0,1-c)}$ or $\mathbb {1}_{[c,1)}$ is solution of Problem (8) when the kernel $\textrm{k}^+$ is considered,
-  $\mathbb {1}_{[0,1-c)}$ or $\mathbb {1}_{[c,1)}$ is solution of Problem (7) when the kernel $\textrm{k}^-$ is considered.

From the proof of Proposition 6.2, we cannot expect to have strict inequalities in (59) if $\alpha$ is only non-decreasing, and thus one cannot expect $\mathcal {S}_0\cup \mathcal {S}_1$ to contain $\mathcal {P}^\textrm{Anti}$ for the kernel $\textrm{k}^+$ or $\mathcal {P}$ for the kernel $\textrm{k}^-$.

### Proof of Proposition 6.2

We assume that $R_0>0$ and $\alpha$ is a strictly increasing function defined on $\Omega =[0, 1)$ such that (49) holds. Without loss of generality, we shall assume that $R_0=1$ unless otherwise specified. We write $R_e^\varepsilon$ for the effective reproduction function associated to the kernel $\textrm{k}^\varepsilon$. We shall also write $\varepsilon a$ for *a* if $\varepsilon =+$ and $-a$ if $\varepsilon =-$. We first rewrite $R_e^\varepsilon$ in two different ways in Sect. 6.4.1. Then, we consider the kernel $\textrm{k}^-$ in Sect. 6.4.2 and the kernel $\textrm{k}^+$ in Sect. 6.4.3.

#### Two expressions of the effective reproduction function

We provide an explicit formula for the function $R_e^\varepsilon$, and an alternative variational formulation, both of which will be needed below.

##### Lemma 6.5

Assume $R_0=1$ and $\alpha$ is a strictly increasing function defined on $\Omega =[0, 1)$ such that (49) holds. We have for $\varepsilon \in \{+, -\}$ and $\eta \in \Delta$:

50 $$\begin{aligned} 2R_e^\varepsilon (\eta )= \int \eta \, \textrm{d} \mu +\varepsilon \int \alpha ^2\, \eta \, \textrm{d} \mu + \sqrt{\left( \int \eta \, \textrm{d} \mu -\varepsilon \int \alpha ^2\, \eta \, \textrm{d} \mu \right) ^2 + 4\varepsilon \left( \int \alpha \, \eta \, \textrm{d} \mu \right) ^2}. \end{aligned}$$

Alternatively, $R_e^\varepsilon (\eta )$ is the solution of the variational problem:

51 $$\begin{aligned} R_e^\varepsilon (\eta ) = \sup _{h\in B^\eta _+} \left( \int _0^1 h\, \eta \, \textrm{d} \mu \right) ^2 + \varepsilon \left( \int _0^1 h\, \alpha \, \eta \, \textrm{d} \mu \right) ^2, \end{aligned}$$

where

$$\begin{aligned} B_+^\eta =\left\{ h\in L^2_+\, :\, \int _0^1 h^2\, \eta \, \textrm{d} \mu = 1\right\} . \end{aligned}$$

The supremum in (51) is reached for the right Perron eigenfunction of $T_{\textrm{k}\eta }$ chosen in  $B_+^\eta$.

##### Proof

We first prove (50). For all $\eta \in \Delta$, the rank of the kernel operator $T_{\textrm{k}^\varepsilon \eta }$ is smaller or equal to 2 and  $\textrm{Im} (T_{\textrm{k}^\varepsilon \eta }) \subset \textrm{Vect}({\mathbb {1}}, \alpha )$. The matrix of $T_{\textrm{k}^\varepsilon \eta }$ in the basis $({\mathbb {1}}, \alpha )$ of the range of $T_{\textrm{k}^\varepsilon \eta }$ is given by:

52 $$\begin{aligned} \begin{pmatrix} \int \eta \, \textrm{d} \mu &{} \int \alpha \, \eta \, \textrm{d} \mu \\ \varepsilon \int \alpha \, \eta \, \textrm{d} \mu &{} \varepsilon \int \alpha ^2\, \eta \, \textrm{d} \mu \end{pmatrix}. \end{aligned}$$

An explicit computation of the spectrum of this matrix yields Equation (50) for its largest eigenvalue.

The variational formula (51) is a direct consequence of general Lemma 6.6 below. $\square$

##### Lemma 6.6

(Variational formula for $R_e$ when $\textrm{k}$ is symmetric) Suppose that $\textrm{k}$ is a symmetric kernel on $\Omega$ with a finite double-norm in $L^2$. Then, we have that for all $\eta \in \Delta$:

53 $$\begin{aligned} R_e(\eta ) = \sup _{h\in B^\eta _+} \, \int _{\Omega \times \Omega } h(x) \eta (x)\, \textrm{k}(x,y) \, h(y) \eta (y) \, \mu (\textrm{d}x) \mu (\textrm{d}y), \end{aligned}$$

where

$$\begin{aligned} B_+^\eta =\left\{ h\in L^2_+\, :\, \int _\Omega h^2\, \eta \, \textrm{d} \mu = 1\right\} . \end{aligned}$$

The supremum in (53) is reached for the right Perron eigenfunction of $T_{\textrm{k}\eta }$ chosen in  $B_+^\eta$.

##### Proof

For a finite measure $\nu$ on $(\Omega , \mathscr {F})$, as usual, we denote by $L^2(\nu )$ the set of measurable real-valued functions *f* such that $\int _\Omega f^2 \textrm{d} \nu <+\infty$ endowed with the usual scalar product, so that $L^2(\nu )$ is an Hilbert space. Let $\eta \in \Delta$. We denote by $\mathcal {T}_{\textrm{k}\eta }$ the integral operator associated to the kernel $\textrm{k}\eta$ seen as an operator on the Hilbert space $L^2(\eta \textrm{d} \mu )$: for $g\in L^2(\eta \textrm{d} \mu )$ and $x\in \Omega$ we have $\mathcal {T}_{\textrm{k}\eta }(g)(x)= \int _\Omega \textrm{k}(x,y)\, \eta (y)\, g(y)\,\mu (\textrm{d}y)$. The operator $\mathcal {T}_{\textrm{k}\eta }$ is self-adjoint and compact since the double-norm of $\textrm{k}$ in $L^2(\eta \textrm{d} \mu )$ is finite. It follows from the Krein-Rutman theorem and the Courant-Fischer-Weyl min-max principle that its spectral radius is given by the variational formula:

$$\begin{aligned} \rho (\mathcal {T}_{\textrm{k}\eta })= \sup _{h\in B^\eta _+} \, \int _{\Omega \times \Omega } h(x) \, \textrm{k}(x,y) \, h(y) \, \eta (x)\mu (\textrm{d}x) \, \eta (y)\mu (\textrm{d}y). \end{aligned}$$

Besides, the set $L^2(\mu )$ is densely and continuously embedded in $L^2( \eta \textrm{d} \mu )$ and the restriction of $\mathcal {T}_{\textrm{k}\eta }$ to $L^2(\mu )$ is equal to $T_{\textrm{k}\eta }$. Thanks to ((Delmas et al. 2021a, Lemmas 2.1 (iii) and 2.2), we deduce that $\rho (T_{\textrm{k}\eta })$ is equal to $\rho (\mathcal {T}_{\textrm{k}\eta })$, which gives (53).

Let $h_0$ be the right Perron eigenfunction of $T_{\textrm{k}\eta }$ chosen such that $h_0\in B_+^\eta$. We get:

$$\begin{aligned} \int _{\Omega \times \Omega } \eta (x)h_0(x)\, \textrm{k}(x,y)\, \eta (y) h_0(y)\, \mu (\textrm{d}x)\mu (\textrm{d}y)= R_e(\eta ) \int _{\Omega } \eta (x)h_0(x)^2\, \mu (\textrm{d}x)=R_e(\eta ). \end{aligned}$$

Thus, the supremum in (53) is reached for $h=h_0$. $\square$

#### The kernel $\textrm{k}^-$

Since $\alpha$ is increasing, we have $\mu (\alpha ^2 = R_0) = 0$ and thus the symmetric kernel $\textrm{k}^-$ is positive $\mu ^{\otimes 2}$-a.s. It follows from Remark 3.1 that $c^\star =0$ and $c_\star =1$, and the strategy ${\mathbb {1}}$ (resp. ) is the only Pareto optimal as well as the only anti-Pareto optimal strategy with cost $c=0$ (resp. $c=1$). Since the kernel $\textrm{k}^-$ is constant degree and symmetric, and the non-zero eigenvalues of $T_{\textrm{k}^-}$ are given by $R_0=1$ and $-\int \alpha ^2\, {\textrm{d}}\mu$, the latter being negative, we deduce from Corollary 5.5 (ii) that $\mathcal {S}^\textrm{uni}\subset \mathcal {P}^\textrm{Anti}$. On the one hand, if $\eta$ is anti-Pareto optimal with the same cost as $\eta ^\textrm{uni}\in \mathcal {S}^\textrm{uni}$, one can use that $R_e^-(\eta )=\int \eta \, \textrm{d}\mu$ (as $R_e^-(\eta ^\textrm{uni})=\int \eta ^\textrm{uni}\, \textrm{d}\mu$) and (50) to deduce that $\eta \in \mathcal {S}^{\bot \alpha }$. On the other hand, if $\eta$ belongs to $\mathcal {S}^{\bot \alpha }$, we deduce from (50) that $R_e(\eta )=\int \eta \, \textrm{d}\mu$, and thus $\eta$ is anti-Pareto optimal. In conclusion, we get $\mathcal {P}^\textrm{Anti}= \mathcal {S}^{\bot \alpha }$.

We now study the Pareto optimal strategies. We first introduce a notation inspired by the stochastic order of real valued random variables: we say that $\eta _1, \eta _2 \in \Delta$ with the same cost are in *stochastic order*, and we write $\eta _1 \le _{\textrm{st}} \eta _2$ if:

54 $$\begin{aligned} \int _0^t \eta _1 \, \textrm{d} \mu \ge \int _0^t \eta _2 \, \textrm{d} \mu \quad \text {for all}\quad t\in [0, 1]. \end{aligned}$$

We also write $\eta _1 <_{\textrm{st}} \eta _2$ if the inequality in (54) is strict for at least one $t\in (0, 1)$. If $\eta _1 <_{\textrm{st}} \eta _2$ and *h* is an increasing bounded function defined on [0, 1), then we have:

55 $$\begin{aligned} \int _\Omega h\, \eta _1 \, \textrm{d} \mu < \int _\Omega h\, \eta _2 \, \textrm{d} \mu . \end{aligned}$$

Let $c\in (0, 1)$ be fixed. Define the vaccination strategies with cost *c*:

56 $$\begin{aligned} \eta _0=\mathbb {1}_{[0,1-c)} \quad \text {and}\quad \eta _{1}= \mathbb {1}_{[c,1)}. \end{aligned}$$

In particular we have $\eta _0<_{\textrm{st}}\eta _{1}$ as $\mu$ has no atom and $\Omega$ as full support. Let $\eta \notin \{\eta _0,\eta _{1}\}$ be a vaccination strategy with cost *c*; necessarily

$$\begin{aligned} \eta _0<_{\textrm{st}}\eta <_{\textrm{st}}\eta _{1}. \end{aligned}$$

We now rewrite the function $R_e^-$ in order to use the stochastic order on the vaccination strategies. We deduce from (50) that:

57 $$\begin{aligned} 4 R_e^-(\eta )=4 \int \eta \, \textrm{d} \mu - H(\eta )^2 \quad \text {with}\quad H(\eta )= \sqrt{\int (1+\alpha )^2\eta \, \textrm{d} \mu } - \sqrt{\int (1-\alpha )^2\eta \, \textrm{d} \mu } . \end{aligned}$$

Then, using that $\alpha$ is increasing and $[-1,1]$-valued, we deduce from (55) (with $h=(1+\alpha )^2$ and $h=-(1-\alpha )^2$) and the definition of *H* in (57) that:

$$\begin{aligned} H(\eta _0)< H(\eta ) < H(\eta _{1}). \end{aligned}$$

This readily implies that $R_e^-(\eta )> \min \left( R_e^-(\eta _0), R_e^-(\eta _{1})\right)$. Thus, among strategies of cost *c*, the only possible Pareto optimal ones are $\eta _0$ and $\eta _{1}$. We deduce that $\mathcal {P}\subset \mathcal {S}_0\cup \mathcal {S}_1$.

#### The kernel $\textrm{k}^+$

Arguing as for $\textrm{k}^-$, we get that $c^\star =0$ and $c_\star =1$, and the strategy ${\mathbb {1}}$ (resp. ) is the only Pareto optimal as well as the only anti-Pareto optimal strategy with cost $c=0$ (resp. $c=1$). Since the kernel $\textrm{k}^+$ is constant degree and symmetric, and the non-zero eigenvalues of $T_{\textrm{k}^+}$ given by $R_0$ and $\int _\Omega \alpha ^2\, \textrm{d}\mu$ are positive, we deduce from Corollary 5.5 (i) that  $\mathcal {S}^\textrm{uni}\subset \mathcal {P}$.

Arguing as in Sect. 6.4.2 for the identification of the anti-Pareto optima based on (50) (with $\varepsilon =+$ instead of $\varepsilon =-$) and using that $\mathcal {S}^\textrm{uni}\subset \mathcal {P}$ (instead of $\mathcal {S}^\textrm{uni}\subset \mathcal {P}^\textrm{Anti}$), we deduce that $\mathcal {P}= \mathcal {S}^{\bot \alpha }$.

We now consider the anti-Pareto optima. Let $c\in (0, 1)$. We first start with some comparison of integrals with respect to the vaccination strategies, with cost *c*, $\eta _0$ and $\eta _{1}$ defined by (56). Let $\eta$ be a strategy of cost *c* not equal to $\eta _0$ or $\eta _{1}$ (recall that a strategy is defined up to the a.s. equality). Consider the monotone continuous non-negative functions defined on [0, 1]:

$$\begin{aligned} \phi _0\, :\, x \mapsto \varphi ^{-1} \left( \int _{[0,x)} \eta \, \textrm{d} \mu \right) , \quad \text {and}\quad \phi _1\, :\, x \mapsto \varphi ^{-1} \left( 1 - \int _{[x,1)} \eta \, \textrm{d}\mu \right) . \end{aligned}$$

Let $i\in \{0,1\}$. Let $\phi _i^{-1}$ denote the generalized left-continuous inverse of $\phi _i$. Note that $\eta (x) \mu (\textrm{d} x)$-a.s., $\phi ^{-1}_i \circ \phi _i(x)=x$. The measure $\eta _i\, \textrm{d} \mu$ is the push-forward of $\eta \, \textrm{d} \mu$ through $\phi _i$, so that for *h* bounded measurable:

58 $$\begin{aligned} \int h \, \eta \, \textrm{d} \mu = \int h_i\, \eta _i\, \textrm{d} \mu \quad \text {with}\quad h_i=h\circ \phi _i^{-1}. \end{aligned}$$

Since $\eta$ is not equal to $\eta _0$ a.s., there exists $x_0 < 1 - c$ such that, $\phi _0(x) = x$ for $x \in [0,x_0]$ and $\phi _0(x) < x$ for $x \in (x_0, 1]$. Thus, we deduce that $\phi _0^{-1}(y) = y$ for all $y \in [0, x_0]$ and $\phi _0^{-1}(y)>y$ for all $y \in (x_0, 1-c]$. Similarly, since $\eta$ is not equal to $\eta _{1}$ almost surely, there exists $x_1 > c$ such that $\phi _1^{-1}(y) = y$ for all $y \in (x_1,1]$ and $\phi _1^{-1}(y) < y$ for all $y \in [c, x_1)$. Since $\alpha$ is increasing and $\mu$ has no atom and full support in $\Omega$, we deduce from from (58), applied to $h\alpha$, that if *h* is a.s. positive bounded measurable, then:

59 $$\begin{aligned} \int h_0\, \alpha \, \eta _0\, \textrm{d} \mu< \int h\, \alpha \, \eta \, \textrm{d} \mu < \int h_1\, \alpha \, \eta _{1}\, \textrm{d}\mu . \end{aligned}$$

Let *h* be the right Perron eigenfunction of $T_{\textrm{k}^+ \eta }$ chosen such that $h\in B_+^\eta$. Since $\textrm{k}^+$ is positive a.s. and thus irreducible with positive spectral radius, we have that *h* is positive a.s. Thanks to Lemma 6.5, we have:

60 $$\begin{aligned} R_e^+(\eta ) = \left( \int h\, \eta \, \textrm{d} \mu \right) ^2 + \left( \int h\, \alpha \, \eta \, \textrm{d} \mu \right) ^2 \quad \text {and}\quad \int h^2 \, \eta \, \textrm{d} \mu =1 . \end{aligned}$$

We deduce from (58) that for $i\in \{0,1\}$:

$$\begin{aligned} \int h\, \eta \, \textrm{d} \mu =\int h_i\, \eta _i\, \textrm{d} \mu \quad \text {and}\quad 1=\int h^2\, \eta \, \textrm{d} \mu =\int h_i^2\, \eta _i\, \textrm{d} \mu . \end{aligned}$$

In particular $h_i$ belongs to $B_+^{\eta _i}$. Using that $h>0$ a.s., we then deduce from (60) and (59) that:

$$\begin{aligned} R_e^+(\eta ) < \max _{i\in \{0,1\}} \left( \int h_i\, \eta _i \, \textrm{d} \mu \right) ^2 + \left( \int h_i\, \alpha \, \eta _i\, \textrm{d} \mu \right) ^2 \le \max _{i\in \{0,1\}} R_e(\eta _i). \end{aligned}$$

We conclude that only $\eta _0$ or $\eta _{1}$ can maximize $R_e^+$ among the strategies of cost $c\in (0,1)$. We deduce that $\mathcal {P}^\textrm{Anti}\subset \mathcal {S}_0\cup \mathcal {S}_1$.

### An example where all parametrizations of the Pareto frontier have an infinite number of discontinuities

The purpose of this section is to give a particular example of kernel on a continuous model where we rigorously prove that the Pareto frontier cannot be greedily parametrized, that is, parametrized by a continuous path in $\Delta$ (as in the fully symmetric circle), and that all the parametrizations have an arbitrary large number of discontinuities (possibly countably infinite).

We keep the setting from Sect. 6.2. Without loss of generality, we assume that $R_0 = 1$, and we consider the kernel $\textrm{k}^-=1- \alpha \otimes \alpha$ on $\Omega =[0, 1)$ endowed with its Lebesgue measure. We know from the previous section that, for any cost, either $\eta _0$ or $\eta _{1}$ are Pareto optimal, and that all other strategies are non-optimal. The idea is then to build an instance of the function $\alpha$ in such a way that for some costs, one must vaccinate “on the left” and for other costs “on the right”.

Let $N \in [\![2, + \infty ]\!]$. Consider an increasing sequence $(x_n, \, n\in [\![0, N]\!])$ such that $x_0 = 1/2$, $x_N = 1$ and $\lim _{n\rightarrow \infty } x_n = 1$ if $N = \infty$. For $0 \le n < N$, let $p_n = x_{n+1} - x_n$ and assume that $p_{n+1} < p_n$ for $n\in [\![0, N[\![$. For $n \ge 1$, let $x_{-n}$ be the symmetric of $x_n$ with respect to 1/2, *i.e.*, $x_{-n} = 1 - x_n$. The function $\alpha$ is increasing piecewise linear defined on (0, 1) by:

61 $$\begin{aligned} \alpha (x) = {\left\{ \begin{array}{ll} 2x - 1, &{}\quad \text {for} \; x \in [x_{2m}, x_{2m + 1}), \\ \\ x - 1 + \frac{x_{2m-1} + x_{2m}}{2} &{}\quad \text {for} \; x \in [x_{2m-1}, x_{2m}). \\ \end{array}\right. } \end{aligned}$$

**Fig. 10:**
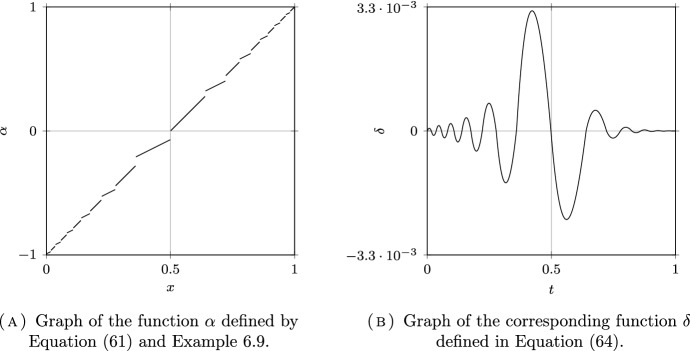
Plots of the functions of interest in Sect. 6.5

See Fig. 10A for an instance of the graph of $\alpha$ given in Example 6.9. Note that for all $n\in [\![0, N[\![$, we have:

62 $$\begin{aligned} \int _{x_{n}}^{x_{n + 1}} \alpha \, \textrm{d} \mu = -\int _{x_{-n-1}}^{x_{-n}} \alpha \, \textrm{d} \mu . \end{aligned}$$

This proves that the integral of $\alpha$ over [0, 1) is equal to 0. Of course, $\sup _{[0,1)} \alpha ^2 = 1 = R_0$. Hence, $\alpha$ satisfies Condition (49).

We recall that a function $\gamma : [0, c_\star ] \mapsto \Delta$ is a parametrization of the Pareto frontier if for all $c\in [0, c_\star ]$ the strategy $\gamma (c)$ is Pareto optimal with cost $C(\gamma (c))=c$. Now we can prove there exists no greedy parametrization of the Pareto frontier of the kernel $\textrm{k}^-$ and even impose an arbitrary large lower bound for the number of discontinuities.

#### Proposition 6.7

Let $N \in [\![2, + \infty ]\!]$. Consider the kernel $\textrm{k}^- = 1 - \alpha \otimes \alpha$ from (48) on $\Omega =[0, 1)$ endowed with its Lebesgue measure, with $\alpha$ given by (61). Then, any parametrization of the Pareto frontier has at least $2N-2$ and at most $20N-2$ discontinuities.

The proof is given at the end of this section, and relies on the following technical lemma based on the comparison of the following monotone paths $\gamma _0$ and $\gamma _1$ from [0, 1] to $\Delta$:

63 $$\begin{aligned} \gamma _0(t) = \mathbb {1}_{[0,t)}, \quad \text {and} \quad \gamma _1(t) = \mathbb {1}_{[1-t,1)}, \quad t \in [0,1] \end{aligned}$$

which parameterizes $\mathcal {S}_0$ and $\mathcal {S}_1$ as $\gamma _0([0, 1])=\mathcal {S}_0$ and $\gamma _1([0, 1])=\mathcal {S}_1$. Notice that strategies $\gamma _0(t)$ and $\gamma _1(t)$ have the same cost $1-t$.

Consider the function $\delta :[0, 1] \rightarrow \mathbb {R}$ which, according to Proposition 6.2, measures the difference between the effective reproduction numbers at the extreme strategies:

64 $$\begin{aligned} \delta (t) = R_e(\gamma _0(t)) - R_e(\gamma _1(t)). \end{aligned}$$

The function $\delta$ is continuous and $\delta (0) = \delta (1) = 0$; see for example Fig. 10B for its graph when $\alpha$ is taken from Example 6.9. We say that $t\in (0, 1)$ is a zero crossing of $\delta$ if $\delta (t)=0$ and there exists $\varepsilon >0$ such that $\delta (t+r)\delta (t-r)<0$ for all $r\in (0, \varepsilon )$. The following result gives some information on the zeros of the function $\delta$.

#### Lemma 6.8

Under the assumptions of Proposition 6.7, the function $\delta$ defined in (64) has at least $2N -2$ zero-crossings in (0, 1) and at most 20*N* zeros in [0, 1]. Besides, if $N = \infty$, 0 and 1 are the only accumulation points of the set of zeros of $\delta$.

#### Proof

Using the explicit representation of $R_e^-$ from Lemma 6.5, see (50) with $\varepsilon =-$, we get the function $\delta$ can be expressed as:

65 $$\begin{aligned} 2 \delta (t) = V_1(t) - V_0(t) + \sqrt{ V_0(t) ^2 - M_0(t)^2} - \sqrt{ V_1(t) ^2 - M_1(t)^2} , \end{aligned}$$

where, as $$\int \alpha \, \textrm{d}\mu =0$$:

$$\begin{aligned} M_0(t) = 2 \int _0^t \!\!\alpha \, \textrm{d} \mu , \,\,\, V_0(t) = t+\int _0^t \!\!\alpha ^2\, \textrm{d} \mu , \, \,\, M_1(t)=M_0(1-t)\,\, \, \text {and} \quad V_1(t) = t+ \int _{1-t}^1 \!\!\alpha ^2 \, \textrm{d} \mu . \end{aligned}$$

Elementary computations give that for all $n\in [\![0, N[\![$:

66 $$\begin{aligned} \int _{x_{n}}^{x_{n + 1}} \alpha (x)^2 \, \textrm{d}x - \int _{x_{-n-1}}^{x_{-n}} \alpha (x)^2 \, \textrm{d} x = \frac{(-1)^n p_n^3}{4}, \end{aligned}$$

where we recall that $p_n=x_{n+1} - x_n$. Hence, we obtain that for all $n \in [\![-N, N ]\!]$:

67 $$\begin{aligned} V_1(x_n) - V_0(x_n) = \frac{1}{4} \sum \limits _{i=\left|\,n\,\right|}^\infty (-1)^i p_i^3. \end{aligned}$$

Since the sequence $(p_n, \, n \in [\![0, N [\![)$ is decreasing, we deduce that the sign of $V_1(x_n) - V_0(x_n)$ alternates depending on the parity of $n\in ]\!]-N, N [\![$: it is positive for odd *n* and negative for even *n*. The same result holds for the numbers $\delta (x_n)$ since $M_0(x_n) =M_1(1-x_n)$ for all $n \in [\![-N, N ]\!]$ according to (62) (use that, with $b>0$, the function $x\mapsto x - \sqrt{x^2 - b^2}$ is decreasing for $x\ge \sqrt{b}$ as its derivative is negative). This implies that $\delta$ has at least $2N-2$ zero-crossings in (0, 1).

We now prove that $\delta$ has at most 20*N* zeros in [0, 1] and that 0 and 1 are the only possible accumulation points of the set of zeros of $\delta$. It is enough to prove that $\delta$ has at most 10 zeros on $[x_n, x_{n+1}]$ for all finite $n\in [\![-N, N [\![$. On such an interval $[x_n, x_{n+1}]$, the function $\alpha$ is a polynomial of degree one. Consider first *n* odd and non-negative, so that for $t\in [x_n, x_{n+1}]$, we get that with $a=1-(x_n+x_{n+1})/2$:

$$\begin{aligned} M_0(t)=2t^2 - 2t + b_1, \quad&V_0(t)=\frac{4}{3} t^3 - 2 t^2 + 2t + b_2, \\ M_1(t)=t^2 -2a t + b_3 , \quad&V_1(t)=- \frac{1}{3} t^3 + a t^2 + (1- a^2) t + b_4, \end{aligned}$$

where $b_i$ are constants. If *t* is a zero of $\delta$, then it is also a zero of the polynomial *P* given by:

$$\begin{aligned} P= 4(V_1-V_0)\left( V_0M_1^2 - V_1M_0^2\right) - \left( M_0^2 - M_1^2\right) ^2. \end{aligned}$$

Since the degree of *P* is exactly 10, it has at most 10 zeros. Thus $\delta$ has at most 10 zeros on $[x_n, x_{n+1}]$. This ends the proof. $\square$

#### Proof of Proposition 6.7

According to Proposition 6.2, the only possible Pareto strategies of cost $c=1-t\in [0, 1]$ are $\gamma _0(t)$ and $\gamma _1(t)$, and only one of them is optimal when $\delta \ne 0$. A zero crossing of the function $\delta$ on (0, 1) therefore corresponds to a discontinuity of any parametrization of the Pareto frontier. We deduce from Lemma 6.8 that in (0, 1) there are at least $2N-2$ and at most $20N- 2$ zeros crossing and thus discontinuities of any parametrization of the Pareto frontier. $\square$

**Fig. 11:**
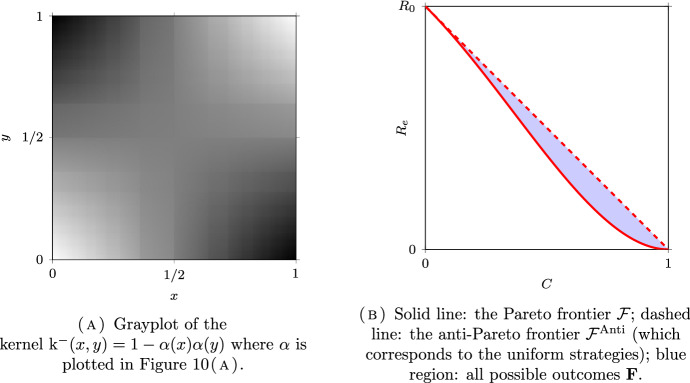
An example of a constant degree kernel operator of rank two

#### Example 6.9

In Fig. 10A, we have represented the function $\alpha$ defined by (61) where:

$$\begin{aligned} x_n = \frac{1}{2} \log _{12}(12(n+1)), \quad 0 \le n \le N=11. \end{aligned}$$

Hence, the mesh $(x_n, \, -N \le n \le N)$ is composed by $2N +1 = 23$ points. The graph of the corresponding function $\delta$ defined in (64) is drawn in Fig. 10B. The grayplot of the kernel $\textrm{k}^-=1- \alpha \otimes \alpha$ is given in Fig. 11A and the associated Pareto and anti-Pareto frontiers are plotted in Fig. 11B.

## Geometric kernels on the sphere

A geometric random graph is an undirected graph constructed by assigning a random point in a latent metric space to each node and by connecting two nodes according to a certain probability that depends on the distance between their latent point. Because of its geometric stucture, this model is appealing for a wide-range of applications such as wireless networks modelling (Hekmat and Van Mieghem 2003), social networks (Hoff et al. 2002) and biological networks (Higham et al. 2008). A geometric random graph model can be represented as a symmetric kernel defined on the latent space (also called *graphon*) according to Lovász (2012).

In this section, we focus our study on the latent space given by the unit sphere. In Sect. 7.1 we present the mathematical model, and give in Sect. 7.2 sufficient conditions on the kernel for uniform strategies to be Pareto or anti-Pareto optimal. Section 7.3 is devoted to the explicit descriptions of the Pareto and anti-Pareto optimal vaccination strategies in the affine case.

### The model

Let $d\ge 2$. Let $\Omega = {\mathbb {S}^{d-1}}$ be the unit sphere of the Euclidean *d*-dimensional space $\mathbb {R}^d$ endowed with the usual Borel $\sigma$-field and the uniform probability measure $\mu$. Let $\langle \cdot , \cdot \rangle$ denote the usual scalar product on $\mathbb {R}^d$ and letdenote the geodesic distance between $x,y\in {\mathbb {S}^{d-1}}$. By symmetry, the distribution on $[-1, 1]$ of the scalar product of two independent uniformly distributed random variables in ${\mathbb {S}^{d-1}}$ is equal to the distribution of the first coordinate of a uniformly distributed unit vector: it is the probability measure on $[-1, 1]$ with density with respect to the Lebesgue measure proportional to the function $w_d$ defined on $[-1, 1]$ by:

$$\begin{aligned} w_d(t)=(1 - t^2)^{(d-3)/2}\, \mathbb {1}_{(-1, 1)}(t). \end{aligned}$$

In particular, we deduce from the Funk-Heck formula (take $n=0$ in (Dai and Xu 2013, Theorem 1.2.9) that for any non-negative measurable function *h* defined on $[-1, 1]$ and $x\in {\mathbb {S}^{d-1}}$, we have:

68 $$\begin{aligned} \int _{{\mathbb {S}^{d-1}}} h(\langle x,y \rangle )\, \mu (\textrm{d} y) = c_d\int _{-1}^ 1 h(t) \, w_d(t) \, \textrm{d}t \quad \text {with}\quad c_d=\frac{\Gamma (\frac{d}{2})}{\Gamma (\frac{d-1}{2}) \sqrt{\pi }} \cdot \end{aligned}$$

We consider a symmetric kernel $\textrm{k}$ on $\mathbb {S}^{d-1}$ corresponding to a geometric random graph model on $\mathbb {S}^{d-1}$, given by:69where $p \, :\, [-1,1] \rightarrow \mathbb {R}_+$ is a measurable function and $f = p \circ \cos \, :\, [0, \pi ] \rightarrow \mathbb {R}_+$. We assume that $\textrm{k}$ has finite double-norm on $L^2$; thanks to (68), this is equivalent to:

70 $$\begin{aligned} \int _{-1}^1 p(t)^2 \, w_d(t) \, \textrm{d}t = \int _0^\pi f(\theta )^2 \sin (\theta )^{d-2} \textrm{d}\theta < \infty . \end{aligned}$$

By symmetry, using that the scalar product and the measure $\mu$ are invariant by rotations, we deduce that the kernel $\textrm{k}$ is a constant degree kernel. According to (45) and using (68), we get that the basic reproduction number is given by:

71 $$\begin{aligned} R_0 = c_d \int _{-1}^{1} p(t) \, w_d(t) \, \textrm{d}t = c_d \int _0^\pi f(\theta ) \sin (\theta )^{d-2}\, \textrm{d}\theta . \end{aligned}$$

By (Dai and Xu 2013, Theorem 1.2.9), the eigenvectors of the symmetric operator $T_\textrm{k}$ on $L^2({\mathbb {S}^{d-1}})$ are the spherical harmonics, and in particular, they don’t depend on the function *p*. We recall the linear subspace of spherical harmonics of degree *n* for $n \in \mathbb {N}$ has dimension $d_n$ given by $d_0=1$ and for $n\in \mathbb {N}^*$:

$$\begin{aligned} d_n = \frac{2n+ d-2}{n+d-2} \left( {\begin{array}{c}n + d - 2\\ n\end{array}}\right) . \end{aligned}$$

The corresponding eigenvalues $(\lambda _n, n\in \mathbb {N})$ are real and given by:

72 $$\begin{aligned} \lambda _n =c_d \int _{-1}^1 p(t) \, \frac{G_n(t)}{ G_n(1) } \, w_d(t) \, \textrm{d}t = c_d \int _0^\pi f(\theta )\, \frac{G_n(\cos (\theta ))}{ G_n(1) } \sin (\theta )^{d-2}\, \textrm{d}\theta , \end{aligned}$$

where $G_n$ is the Gegenbauer polynomial of degree *n* and parameter $(d-2)/2$ (see (Dai and Xu 2013, Section B.2) with $G_n=C_n^{(d-1)/2}$). We simply recall that $G_0={\mathbb {1}}$ and that for $d=2$, the Gegenbauer polynomials are, up to a multiplicative constant, the Chebyshev polynomials of the first kind:

$$\begin{aligned} G_n(\cos (\theta ))=\frac{2}{n} \, \cos (n\theta ) \quad \text {for }\theta \in [0, \pi ]\text { and }n\in \mathbb {N}^*; \end{aligned}$$

and that for $d\ge 3$, $r\in (-1,1)$ and $\theta \in [0, \pi ]$:

$$\begin{aligned} \sum _{n=0}^\infty r^n G_n(\cos (\theta ))=(1+r^2 - 2r \cos (\theta ))^{-(d-2)/2} \quad \text {and}\quad G_n(1)=\left( {\begin{array}{c}n+d-3\\ n\end{array}}\right) \quad \text {for~}n\in \mathbb {N}^*. \end{aligned}$$

Thus, if $\lambda \ne 0$ is an eigenvalue of $T_\textrm{k}$, then its multiplicity is the sum of all the dimensions $d_n$ such that $\lambda _n = \lambda$. The eigenvalue $R_0$ (associated to the eigenvector ${\mathbb {1}}$ which is the spherical harmonic of degree 0) is in fact simple according to the next Lemma.

#### Lemma 7.1

Let $\textrm{k}$ be a kernel on ${\mathbb {S}^{d-1}}$ given by (69), with finite double-norm and such that $R_0>0$. Then the kernel $\textrm{k}$ is constant degree and irreducible, and its eigenvalue $R_0$ is simple.

#### Proof

The kernel $\textrm{k}$ is trivially a constant degree kernel. Since $d_0=1$, we only need to prove that $\lambda _n < \lambda _0 = R_0$ for all $n \in \mathbb {N}^*$ to get that $R_0$ is simple, and then use Lemma 5.12 to get that $\textrm{k}$ is irreducible.

According to (Abramowitz and Stegun 1972, Equation 22.14.2) or (Atkinson and Han 2012, Section 3.7.1), we get that $|G_n(t)|\le G_n(1)$ for $t\in [-1, 1]$. Since $G_n$ is a polynomial, the inequality is strict for a.e. $t\in [-1, 1]$. Using (72), we obtain that $\lambda _n < \lambda _0$ for all $n \in \mathbb {N}^*$. $\square$

#### Example 7.2

*(The circle: d = 2)* In case $d = 2$, we identify the circle $\mathbb {S}^1$ with $\Omega =\mathbb {R}/{2\pi \mathbb {Z}}$ and the scalar product $\langle \theta ,\theta ' \rangle =\cos (\theta -\theta ')$. The kernel $\textrm{k}$ from (69) is the convolution kernel given by $k(\theta , \theta ')=p (\cos (\theta -\theta '))=f(\theta -\theta ')$, where *f* is symmetric non-negative and $2\pi$ periodic and its restriction to $[0, \pi ]$ is square integrable. Then, we can consider the development in $L^2([0, \pi ])$ of *f* as a Fourier series:

73 $$\begin{aligned} f(\theta ) = \sum _{n = 0}^\infty a_n(f) \cos (n \theta ), \quad \theta \in [0, \pi ], \end{aligned}$$

where:

74 $$\begin{aligned} a_0(f) = \frac{1}{\pi } \int _0^\pi f(\theta ) \, \textrm{d} \theta \quad \text {and}\quad a_n(f) = \frac{2}{\pi } \int _0^\pi \cos (n \theta ) f(\theta ) \, \textrm{d} \theta \quad \text {for}\quad n \ge 1. \end{aligned}$$

It follows from Equation (73) that the kernel has the following decomposition in $L^2([0, 2\pi )^2)$:

75 $$\begin{aligned} \textrm{k}(\theta ,\theta ') = a_0(f) + \sum _{n = 1}^\infty a_n(f) \, \big (\cos (n \theta ) \cos (n \theta ') + \sin (n \theta ) \sin (n \theta ')\big ), \quad \theta , \theta ' \in [0, 2\pi ).\nonumber \\ \end{aligned}$$

Assume that $a_0(f)>0$, that is, *f* is non-zero. Then, the spectral radius $R_0=a_0(f)$ is an eigenvalue with multiplicity one associated to the eigenfunction ${\mathbb {1}}$ (and thus $\textrm{k}$ is a constant degree kernel). The other eigenvalues are given by $\lambda _{n} = a_n(f)/2$ for all $n \ge 1$ and, when non zero and distinct, have multiplicity 2.

### Sufficient condition for convexity or concavity

We would like to provide conditions on the function *f* or *p* that ensure that the eigenvalues $(\lambda _n, \, n \ge 1)$ given by (72) of the operator $T_\textrm{k}$ with the kernel $\textrm{k}$ defined by (69) are all non-negative or all non-positive so that $R_e$ is convex or concave according to Corollary 5.5. Schoenberg’s theorem, see (Dai and Xu 2013, Theorem 14.3.3) or (Gneiting 2013, Theorem 1), characterizes the continuous function *f* such that the kernel $\textrm{k}$ is positive semi-definite (and thus the eigenvalues $(\lambda _n, \, n \ge 1)$ are all non-negative) as those with non-negative Gegenbauer coefficients: $f=\sum _{n=0}^\infty a_n \, G_n$, where the convergence is uniform on $[-1, 1]$, with $a_n\ge 0$ for all $n\in \mathbb {N}$ and $\sum _{n=0}^\infty a_n \, G_n(1)$ finite. When $d=2$, this corresponds to the Böchner theorem. We refer to Gneiting (2013) and references therein for some characterization of functions *f* such that the kernel $\textrm{k}$ from (69) is definite positive. We end this section with some examples.

#### Example 7.3

We give an elementary example in the setting of Example 7.2 when $d=2$. Set

$$\begin{aligned} f_+(\theta )= (\pi - \theta )^2 \quad \text {and}\quad f_-(\theta )= \pi ^2- (\pi - \theta )^2 \quad \text {for~}\theta \in [0, \pi ]. \end{aligned}$$

We can compute the Fourier coefficients of $f_+$ and $f_-$ as:

$$\begin{aligned} (\pi - \theta )^2 = \frac{\pi ^2}{3} + \sum _{n = 1}^\infty \frac{4}{n^2} \cos (n \theta ), \quad \theta \in [0,\pi ]. \end{aligned}$$

Using Corollary 5.5 and (Delmas et al. 2021a, Theorem 4.10), we deduce that the function $R_e$ associated to the convolution kernel $\textrm{k}=f_+\circ \delta$ is convex and $\mathcal {S}^\textrm{uni}\subset \mathcal {P}$; whereas the function $R_e$ associated to the convolution kernel $\textrm{k}=f_-\circ \delta$ is concave and $\mathcal {S}^\textrm{uni}\subset \mathcal {P}^\textrm{Anti}$.

#### Example 7.4

*(Kernel from a completely monotone function)* Let $\varphi$ be a continuous non-negative function defined on $\mathbb {R}_+$, such that $\varphi$ is completely monotone, that is, $\varphi$ is infinitely differentiable on $(0, +\infty )$ and $(-1)^n \varphi ^{(n)} \ge 0$ on $(0,+\infty )$ for all $n \ge 1$. Using (Gneiting 2013, Theorem 7), we get that the geometric kernel $\textrm{k}= f\circ \delta$ on ${\mathbb {S}^{d-1}}$, with $d=2$, where $f = \varphi _{[0,\pi ]}$ is positive definite (thus all the eigenvalues of $T_\textrm{k}$ are non-negative). Thanks to Corollary 5.5 and (Delmas et al. 2021a, Theorem 4.10), we deduce that $R_e$ is convex and the uniform strategies are Pareto optimal: $\mathcal {S}^\textrm{uni}\subset \mathcal {P}$.

#### Example 7.5

*(Kernel from a Bernstein function)* Let $\varphi$ be a Bernstein function, that is a non-negative $C^1$ function defined on $\mathbb {R}_+$ such that $\varphi ^{(1)}$ is completely monotone. Assume furthermore that $\sup _{\mathbb {R}_+} \varphi < \infty$. This gives that the function $t \mapsto (\sup _{\mathbb {R}_+} \varphi ) - \varphi (t)$ defined on $\mathbb {R}_+$ is continuous non-negative and completely monotone. Consider the geometric kernel $\textrm{k}= f\circ \delta$ on ${\mathbb {S}^{d-1}}$, with $d=2$, where $f = \varphi _{[0,\pi ]}$. We deduce from (Gneiting 2013, Theorem 7), see also the previous example, that all the eigenvalues of the integral operator $T_\textrm{k}$, but for $R_0$, are non-positive. Then, using Corollary 5.5 and (Delmas et al. 2021a, Theorem 4.10), we get that $R_e$ is concave and the uniform strategies are anti-Pareto optimal: $\mathcal {S}^\textrm{uni}\subset \mathcal {P}^\textrm{Anti}$.

#### Example 7.6

*(Kernel from a power function)* Let $m \ge 1$ be an integer and $\theta _0 \ge \pi$ a real number. Using (Gneiting 2013, Lemma 4), we get that for $f(\theta ) = (\theta _0 - \theta )^m$, $R_e$ is convex and the uniform vaccination strategies are Pareto optimal; and that for $f(\theta ) = \theta _0^m - (\theta _0 - \theta )^m$, $R_e$ is concave and the uniform strategies are anti-Pareto optimal.

#### Example 7.7

*(The function p is a power series)* According to (Gneiting 2013, Theorem 1), if the function *p* can be written as $p(t)=\sum _{n\in \mathbb {N}} b_n \, t^n$ with $b_n$ non-negative and $\sum _{n\in \mathbb {N}} b_n$ finite, then, for all $d\ge 2$, the kernel $\textrm{k}$ defined by (69) on ${\mathbb {S}^{d-1}}$ is semi-definite positive (and definite positive if the coefficients $b_n$ are positive for infinitely many even and infinitely many odd integers *n*), and thus the function $R_e$ is convex and the uniform vaccination strategies are Pareto optimal thanks to Corollary 5.5 and (Delmas et al. 2021a, Theorem 4.10).

#### Example 7.8

*(The kernel is a power of the metric)* Consider the function $p(t)=2^{\nu /2} |1-t|^{\nu /2}$, with $\nu > (1-d)/2$, so that condition (70) holds. This corresponds to the kernel $\textrm{k}(x,y)=|x-y|^\nu$ which is a power of the distance between *x* and *y*. According to (Atkinson and Han 2012, Section 3.7.1) and Equation (3.74) therein, for $n\ge 1$, the eigenvalues $\lambda _n$ have the same sign as $\prod _{k=0}^{n-1} (-\nu +2k)$. So, we deduce that for $\nu \in ((1-d)/2, 0)$ all the eigenvalues are positive and thus $R_e$ is convex and the uniform vaccination strategies are Pareto optimal; and for $\nu \in (0, 2)$ all the eigenvalues (but $\lambda _0=R_0>0$) are negative and thus $R_e$ is concave and the uniform strategies are anti-Pareto optimal. The latter case is also a consequence of (Gneiting 2013, Theorem 1), whereas the former case is not a direct consequence of (Gneiting, 2013, Theorem 1) as $\sum _{n\in \mathbb {N}} b_n$ is not finite when $\nu$ is negative.

### The affine model

Recall $\Omega = {\mathbb {S}^{d-1}}\subset \mathbb {R}^d$, with $d\ge 2$, is endowed with the uniform probability measure $\mu$. In this section, we suppose that the model is affine, that is, the kernel $\textrm{k}$ given by (69), *i.e.* , has a linear envelope:

$$\begin{aligned} p(t)=a + b t \quad \text {for}\quad t\in [-1, 1]. \end{aligned}$$

The kernel $\textrm{k}$ being non-negative non-constant with $R_0>0$ is equivalent to the condition $a\ge |b| > 0$ on the parameter (*a*, *b*). This model corresponds to $f(\theta ) = a + b \cos (\theta )$ for $\theta \in [0, \pi ]$. Since the Gegenbauer polynomials $(G_n, n\in \mathbb {N})$ are orthogonal with respect to the measure $w_d(t)\, \textrm{d} t$, we easily deduce from (72) that the non-zero eigenvalues of the integral operator $T_\textrm{k}$ are $R_0=a$ (with multiplicity $d_0=1$) and $\lambda _1= b/d$ (with multiplicity $d_1=d$).

For $x\in {\mathbb {S}^{d-1}}$ and $t\in [-1,1]$, we consider the following balls centered at *x*:

$$\begin{aligned} B(x,t)=\{y\in {\mathbb {S}^{d-1}}\, :\, \langle x,y \rangle \ge t\}. \end{aligned}$$

Recall that strategies are defined up to the a.s. equality. We consider the following sets of extremal strategies, for $x\in {\mathbb {S}^{d-1}}$:

$$\begin{aligned} \mathcal {S}^{\textrm{balls}}= \left\{ \mathbb {1}_{B(x,t)}\, :\, x\in {\mathbb {S}^{d-1}}, \, t \in [-1,1]\right\} , \end{aligned}$$

as well as the following set of strategies which contains the set of uniform strategies $\mathcal {S}^\textrm{uni}=\{ t{\mathbb {1}}\, :\, t\in [0,1]\}$:

$$\begin{aligned} \mathcal {S}^{\bot \textrm{id}}=\left\{ \eta \in \Delta \, :\, \int _{\mathbb {S}^{d-1}}x \, \eta (x) \, \mu (\textrm{d} x) =0\right\} . \end{aligned}$$

#### Proposition 7.9

Let $a\ge |b|>0$ and the kernel $\textrm{k}$ on ${\mathbb {S}^{d-1}}$, with $d\ge 2$, be given by:

$$\begin{aligned} \textrm{k}(x,y)=a+ b \langle x,y \rangle . \end{aligned}$$

(i) **The case** $b>0$. A strategy is Pareto optimal if and only if it belongs to $\mathcal {S}^{\bot \textrm{id}}$. In particular, for any $c\in [0,1]$, the strategy $(1-c){\mathbb {1}}$ costs *c* and is Pareto optimal. The anti-Pareto optimal strategies are $\mathbb {1}_{B(x,t)}$ for $x\in {\mathbb {S}^{d-1}}$ and $t\in [-1,1]$. In other words:
  $$\begin{aligned} \mathcal {P}=\mathcal {S}^{\bot \textrm{id}}\quad \text {and}\quad \mathcal {P}^\textrm{Anti}= \mathcal {S}^{\textrm{balls}}. \end{aligned}$$
(ii) **The case** $b<0$. A strategy is anti-Pareto optimal if and only if it belongs to $\mathcal {S}^{\bot \textrm{id}}$. In particular, for any $c\in [0,1]$, the strategy $(1-c){\mathbb {1}}$ costs *c* and is anti-Pareto optimal. The Pareto optimal strategies are $\mathbb {1}_{B(x,t)}$ for $x\in {\mathbb {S}^{d-1}}$ and $t\in [-1,1]$. In other words:
  $$\begin{aligned} \mathcal {P}^\textrm{Anti}= \mathcal {S}^{\textrm{balls}}\quad \text {and}\quad \mathcal {S}^\textrm{uni}=\mathcal {S}^{\bot \textrm{id}}. \end{aligned}$$

In both cases, we have $c_\star =1$ and $c^\star =0$.

#### Example 7.10

**Fig. 12:**
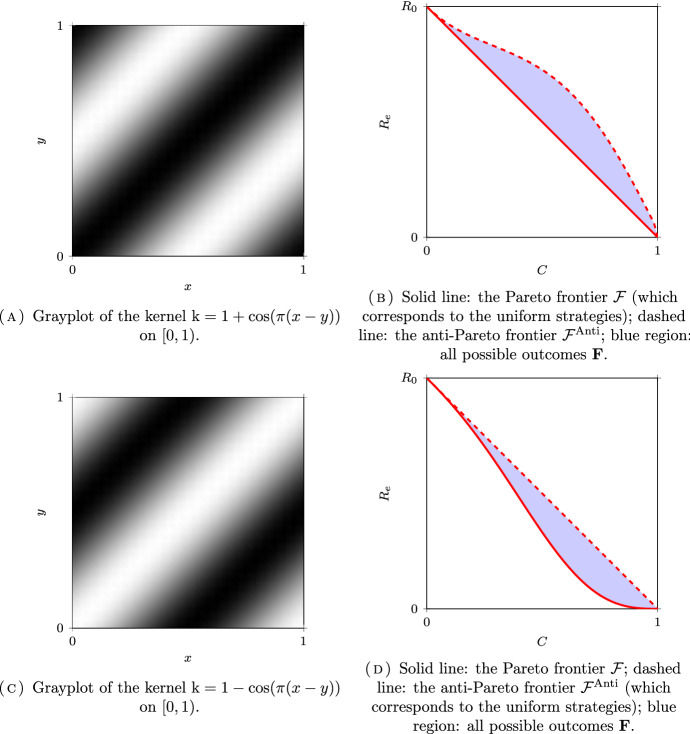
Two examples of a geometric kernel on the circle $\mathbb {R}\setminus \mathbb {Z}$

We consider the kernel $\textrm{k}=1 +b \langle \cdot , \cdot \rangle$ on the sphere ${\mathbb {S}^{d-1}}$, with $d=2$. This model has the same Pareto and anti-Pareto frontiers as the equivalent model given by $\Omega = [0,1)$ endowed with the Lebesgue measure and the kernel $(x,y) \mapsto 1 + b \cos (\pi (x-y))$, where the equivalence holds in the sense of (Delmasetal. 2021b, Section 7), with an obvious deterministic coupling $\theta \mapsto \exp (2i\pi \theta )$. We provide the Pareto and anti-Pareto frontiers in Fig. 12 with $b=1$ (top) and with $b=-1$ (bottom).

#### Proof

The proof of Proposition 7.9 is decomposed in four steps. *Step 1:* $R_e(\eta )$ is the eigenvalue of a $2\times 2$ matrix $M(\eta )$. Without loss of generality, we shall assume that $R_0=a=1$. Since $\textrm{k}$ is positive a.s., we deduce that $c_\star =1$ and $c^\star =0$ thanks to Lemma 3.1; and the strategy ${\mathbb {1}}$ (resp. ) is the only Pareto optimal as well as the only anti-Pareto optimal strategy with cost 0 (resp. 1). So we shall only consider strategies $\eta \in \Delta$ such that $C(\eta )\in (0, 1)$.

Let $z_0\in {\mathbb {S}^{d-1}}$. Write $b=\varepsilon \lambda ^2$ with $\varepsilon \in \{-1,+1\}$ and $\lambda \in (0, 1]$, and define the function $\alpha$ on ${\mathbb {S}^{d-1}}$ by:

$$\begin{aligned} \alpha =\lambda \, \langle \cdot , z_0 \rangle . \end{aligned}$$

Let $\eta \in \Delta$ with cost $c\in (0, 1)$. As $c_\star =1> C(\eta )$, we get that $R_e(\eta )>0$. We deduce from the special form of the kernel $\textrm{k}$ that the eigenfunctions of $T_{\textrm{k}\eta }$ are of the form $\zeta +\beta \lambda \langle \cdot , y \rangle$ with $\zeta , \beta \in \mathbb {R}$ and $y\in {\mathbb {S}^{d-1}}$. Since $R_e(\eta )>0$, the right Perron eigenfunction, say $h_\eta$, being non-negative, can be chosen such that $h_\eta =1+\beta _\eta \lambda \langle \cdot , y_\eta \rangle$ with $\beta _\eta \ge 0$ and $\beta _\eta \lambda \le 1$. Up to a rotation on the vaccination strategy, we shall take $y_\eta =z_0$, that is:

$$\begin{aligned} h_\eta =1+ \beta _\eta \, \alpha . \end{aligned}$$

From the equality $R_e(\eta ) h_\eta = T_{\textrm{k}\eta } h_\eta$, we deduce that:

76 $$\begin{aligned} R_e(\eta )&= \int _{\mathbb {S}^{d-1}}\eta (y) \, \mu ( \textrm{d}y) + \beta _\eta \,\lambda \int _{\mathbb {S}^{d-1}}\eta (y) \, \langle y, z_0 \rangle \, \mu (\textrm{d} y),\end{aligned}$$

77 $$\begin{aligned} \beta _\eta R_e(\eta ) \langle \cdot , z_0 \rangle&=\varepsilon \lambda \int _{\mathbb {S}^{d-1}}\eta (y) \, \langle \cdot , y \rangle \, \mu ( \textrm{d}y)+ \beta _\eta \, \varepsilon \lambda ^2\int _{\mathbb {S}^{d-1}}\eta (y) \, \langle \cdot , y \rangle \langle y, z_0 \rangle \, \mu ( \textrm{d}y) . \end{aligned}$$

Evaluating the latter equality at $x=z_0$, we deduce that $R_e(\eta )$ is a positive eigenvalue of the matrix $M(\eta )$ associated to the eigenvector $(1, \beta _\eta )$, where:

78 $$\begin{aligned} M(\eta )= \begin{pmatrix} \int \eta \, \textrm{d} \mu &{} \int \alpha \,\eta \, \textrm{d} \mu \\ \varepsilon \int \alpha \,\eta \, \textrm{d} \mu &{} \varepsilon \int \alpha ^2\, \eta \, \textrm{d} \mu \\ \end{pmatrix}. \end{aligned}$$

We end this step by proving the following equivalence:

79 $$\begin{aligned} \beta _\eta =0 \Longleftrightarrow \int \alpha \,\eta \, \textrm{d} \mu = 0. \end{aligned}$$

Indeed, if $\beta _\eta =0$, then the vector (1, 0) is an eigenvector of $M(\eta )$ associated to the eigenvalue $R_e(\eta )$. We deduce from (78) that $\int \alpha \,\eta \, \textrm{d} \mu = 0$. Conversely, if $\int \alpha \,\eta \, \textrm{d} \mu = 0$, then the matrix $M(\eta )$ is diagonal with eigenvalues $\int \eta \, \textrm{d}\mu$ and $\int \alpha ^2\, \eta \, \textrm{d} \mu$. As $\alpha ^2\le 1$ with strict inequality on a set of positive $\mu$-measure, we deduce that:

80 $$\begin{aligned} \int \eta \, \textrm{d}\mu > \int \alpha ^2\, \eta \, \textrm{d} \mu . \end{aligned}$$

Since $(1, \beta _\eta )$ is an eigenvector of $M(\eta )$, this implies that $\beta _\eta =0$. This proves (79).

*Step 2:* $R_e(\eta )$ is the spectral radius of the matrix $M(\eta )$, that is, $R_e(\eta )=\rho (M(\eta ))$. We first consider the case $\varepsilon =-1$. Since $\alpha$ is non constant as $\lambda >0$, we deduce from the Cauchy-Schwarz inequality, that the determinant of $M(\eta )$ is negative. As $c_\star =1$ a.s., we deduce that $R_e(\eta )>0$, and thus the other eigenvalue is negative. Since $\alpha ^2\le 1$, the trace of $M(\eta )$ is non-negative, thus $R_e(\eta )$ is the spectral radius of the matrix $M(\eta )$.

We now consider the case $\varepsilon =+1$. Let $\eta ^\textrm{uni}$ be the uniform strategy with the same cost as $\eta$. Thanks to (76), we get $R_e(\eta ^\textrm{uni})=\int \eta ^\textrm{uni}\, \textrm{d}\mu =\int \eta \, \textrm{d}\mu$. Since the non-zero eigenvalues of $T_\textrm{k}$, that is, 1 and $\lambda ^2/d$, are positive, we deduce from Corollary 5.5 (i), that the uniform strategies are Pareto optimal ($\mathcal {S}^\textrm{uni}\subset \mathcal {P}$), so we have:

$$\begin{aligned} R_e(\eta )\ge R_e(\eta ^\textrm{uni})=\int \eta \, \textrm{d} \mu . \end{aligned}$$

We deduce from (76) that $\beta _\eta \int \alpha \,\eta \, \textrm{d} \mu \ge 0$.

On the one hand, if $\beta _\eta \int \alpha \,\eta \, \textrm{d} \mu = 0$, then, by (79), the matrix $M(\eta )$ is diagonal. Using (80), we obtain that $R_e(\eta )=\rho (M(\eta ))$. On the other hand, if $\beta _\eta \int \alpha \,\eta \, \textrm{d} \mu >0$, then the matrix $M(\eta )$ has positive entries. Since the eigenvector $(1, \beta _n)$ also has positive entries, it is the right Perron eigenvector and the corresponding eigenvalue is the spectral radius of $M(\eta )$, that is, $R_e(\eta )=\rho (M(\eta ))$. To conclude, the equality $R_e(\eta )=\rho (M(\eta ))$ holds in all cases.

*Step 3:* $R_e(\eta )=\int \eta \, \textrm{d}\mu \Longleftrightarrow \eta \in \mathcal {S}^{\bot \textrm{id}}$. Let $\eta \in \Delta$ such that $R_e(\eta )=\int \eta \, \textrm{d}\mu$. We deduce from (76) that $\beta _\eta \int \alpha \,\eta \, \textrm{d} \mu = 0$. Thanks to (79), this implies that $\beta _\eta =0$. Using (77), we obtain that $\int y\eta (y) \, \mu (\textrm{d} y)=0$ and thus $\eta \in \mathcal {S}^{\bot \textrm{id}}$. Clearly if $\eta \in \mathcal {S}^{\bot \textrm{id}}$, we deduce from (76) that $R_e(\eta )=\int \eta \, \textrm{d} \mu$.

As a consequence and since $\mathcal {S}^\textrm{uni}\subset \mathcal {S}^{\bot \textrm{id}}$, we deduce from Corollary 5.5 that if $\varepsilon =+1$, then $\mathcal {S}^\textrm{uni}\subset \mathcal {P}$ and thus $\mathcal {P}=\mathcal {S}^{\bot \textrm{id}}$; and that if $\varepsilon =-1$, then $\mathcal {S}^\textrm{uni}\subset \mathcal {P}^\textrm{Anti}$ and thus $\mathcal {P}^\textrm{Anti}=\mathcal {S}^{\bot \textrm{id}}$.

*Step 4:* A relation with the constant degree symmetric kernels of rank two from Sect. 6. This step is in the spirit of (Delmas et al. 2021b, Section 7) on coupled models. Let *X* be a uniform random variable on ${\mathbb {S}^{d-1}}$. Let $\Omega _0=[-1,1]$ endowed with the probability measure $\mu _0(\textrm{d} t)= c_d\, w_d(t)\, \textrm{d} t$, and set $\Delta _0$ the set of [0, 1]-valued measurable functions defined on $\Omega _0$. According to (Kallenberg 2021, Theorem 8.9), there exists $\eta _0 \in \Delta _0$ such that:

81 $$\begin{aligned} \eta _0(\langle X, z_0 \rangle )=\mathbb {E}\left[ \eta (X)\, |\, \langle X, z_0 \rangle \right] \quad \text {a.s.} \end{aligned}$$

Set $\alpha _0=\lambda t$, and define the matrix:

$$\begin{aligned} M_0(\eta _0)= \begin{pmatrix} \int _{\Omega _0} \eta _0 \, \textrm{d} \mu _0 &{} \int _{\Omega _0} \alpha _0\,\eta _0 \, \textrm{d} \mu _0\\ \varepsilon \int _{\Omega _0} \alpha _0\,\eta _0 \, \textrm{d} \mu _0 &{} \varepsilon \int _{\Omega _0} \alpha _0^2\, \eta _0 \, \textrm{d} \mu _0\\ \end{pmatrix}. \end{aligned}$$

By construction of $\eta _0$, we have $M_0(\eta _0)=M(\eta )$. Thanks to Sect. 6, see Lemma 6.5 (but for the fact that $\Omega$ therein in replaced by $[-1, 1]$), we get that $M_0(\eta _0)$ is exactly the matrix in (52), and thus the spectral radius of $M_0(\eta _0)$ is the effective reproduction number of the model associated to the constant degree symmetric kernel of rank two $\textrm{k}_0^\varepsilon =1+ \varepsilon \alpha _0\otimes \alpha _0$ given in (48) (with $\Omega$, $\mu$, $\alpha$ replaced by $\Omega _0$, $\mu _0$ and $\alpha _0$). We deduce that: if $\eta$ is Pareto or anti-Pareto optimal for the model $({\mathbb {S}^{d-1}}, \mu , \textrm{k})$ then so is $\eta _0$ for the model $(\Omega _0, \mu _0, \textrm{k}_0^\varepsilon )$; and if $\eta _0$ is Pareto or anti-Pareto optimal for the model $(\Omega _0, \mu _0, \textrm{k}_0^\varepsilon )$, so is any strategy $\eta$ such that (81) holds.

We first consider the case $\varepsilon =+1$. According to Proposition 6.2, we get that the anti-Pareto optimal strategies are $\eta _0=\mathbb {1}_{[-1, t)}$ or $\eta _0=\mathbb {1}_{[-t, 1)}$ for a given cost *c* (with *t* uniquely characterized by *c*). Using that $0\le \eta \le 1$, we deduce that the only possible choice for $\eta$ such that (81) holds is to take $\eta =\mathbb {1}_{B(-z_0,t)}$ or $\eta =\mathbb {1}_{B(-z_0,t)}$. Since $z_0$ was arbitrary, we get that the only possible anti-Pareto optimal strategies belong to $\mathcal {S}^{\textrm{balls}}$. Notice that anti-Pareto optimal strategies exist for all cost $c\in [0,1]$ as $\textrm{k}>0$ a.s., see Lemma 3.1 and (Delmas et al. 2021b, Section 5.4) for irreducible kernels, loss function $R_e$ and uniform cost function *C* given by (28). Since the set of anti-Pareto optimal strategies is also invariant by rotation, we deduce that $\mathcal {P}^\textrm{Anti}=\mathcal {S}^{\textrm{balls}}$.

The case $\varepsilon =-1$ is similar and thus $\mathcal {P}=\mathcal {S}^{\textrm{balls}}$ in this case. (Note that the irreducibility of the kernel $\textrm{k}$ is only used in (Delmas et al. 2021b, Lemma 5.13) for the study of anti-Pareto frontier.) $\square$

## Footnotes

1 The algorithm is described in Hadka and Reed (2013); we use the version coded in the BlackBoxOptim package for the Julia programming language.
